# Large‐scale phosphomimetic screening identifies phospho‐modulated motif‐based protein interactions

**DOI:** 10.15252/msb.202211164

**Published:** 2023-05-23

**Authors:** Johanna Kliche, Dimitriya Hristoforova Garvanska, Leandro Simonetti, Dilip Badgujar, Doreen Dobritzsch, Jakob Nilsson, Norman E Davey, Ylva Ivarsson

**Affiliations:** ^1^ Department of Chemistry, BMC Uppsala University Uppsala Sweden; ^2^ Faculty of Health and Medical Sciences, Novo Nordisk Foundation Center for Protein Research University of Copenhagen Copenhagen Denmark; ^3^ Division of Cancer Biology The Institute of Cancer Research London UK

**Keywords:** clathrin, phage display, phosphomimetic mutation, phosphorylation, protein–protein interactions, Methods & Resources, Post-translational Modifications & Proteolysis, Proteomics

## Abstract

Phosphorylation is a ubiquitous post‐translation modification that regulates protein function by promoting, inhibiting or modulating protein–protein interactions. Hundreds of thousands of phosphosites have been identified but the vast majority have not been functionally characterised and it remains a challenge to decipher phosphorylation events modulating interactions. We generated a phosphomimetic proteomic peptide‐phage display library to screen for phosphosites that modulate short linear motif‐based interactions. The peptidome covers ~13,500 phospho‐serine/threonine sites found in the intrinsically disordered regions of the human proteome. Each phosphosite is represented as wild‐type and phosphomimetic variant. We screened 71 protein domains to identify 248 phosphosites that modulate motif‐mediated interactions. Affinity measurements confirmed the phospho‐modulation of 14 out of 18 tested interactions. We performed a detailed follow‐up on a phospho‐dependent interaction between clathrin and the mitotic spindle protein hepatoma‐upregulated protein (HURP), demonstrating the essentiality of the phospho‐dependency to the mitotic function of HURP. Structural characterisation of the clathrin‐HURP complex elucidated the molecular basis for the phospho‐dependency. Our work showcases the power of phosphomimetic ProP‐PD to discover novel phospho‐modulated interactions required for cellular function.

## Graphical abstract

A new phosphomimetic proteomic peptide phage library is used to screen for phospho‐modulated interactions between short linear motifs and protein domains. Follow‐up analyses show that S839 phosphorylation of HURP is required for interaction with clathrin and mitotic function.

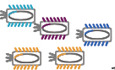

## Introduction

Protein phosphorylation is a commonly occurring post‐translational modification, which provides the cell with an effective mechanism to transmit information on the cell state encoded by kinase and phosphatase activity to the rest of the proteome (Ubersax & Ferrell, 2007; Kliche & Ivarsson, 2022). Phosphorylation occurs most frequently on serine, threonine and tyrosine residues, and the addition of an acidic phosphate moiety can have a significant impact on a protein–protein interaction (PPI; Betts *et al*, 2017). Phosphorylation has also been suggested to occur on histidine (Hunter, 2022), although its importance to mammalian signalling has been questioned (Leijten *et al*, 2022). Phospho‐modulated interactions play a critical role in various cellular processes, orchestrating metabolic and cellular fates, mediating signalling and transport, and regulating cytoskeletal dynamics (Braun & Gingras, 2012; Kliche & Ivarsson, 2022). Large‐scale mass spectrometry‐based proteomic analysis has provided evidence for nearly 300,000 phosphosites (PhosphoSitePlus, Dec 2021; Hornbeck *et al*, 2015, 2019). There is thus compelling evidence for the abundance of phospho‐modulated cellular functions. Several experimental and bioinformatical approaches have been developed to pinpoint key functional phosphosites, including time correlation between kinase activation and the event of phosphorylation (Kanshin *et al*, 2015), evolutionary conservation (Landry *et al*, 2009) and a combination of intrinsic features of phosphosites, such as disorder score and associated kinases (Xiao *et al*, 2016). However, the function of the majority of identified phosphosites is yet to be established.

Phosphorylation frequently regulates interactions mediated by short linear motifs (SLiMs), a subclass of protein interaction modules commonly found in the intrinsically disordered regions of the proteome and exemplified by their compact binding interfaces (typically 3–4 significantly buried residues; Davey *et al*, 2012; Tompa *et al*, 2014). Examples of such phospho‐regulation have been manually curated and compiled in the switches. ELM database (Van Roey *et al*, 2013, Kumar *et al*, 2022). The effects of phosphorylation on binding can range broadly. Phosphosites located within or in close proximity of binding motifs can act as binary on/off switches, as well as fine‐tune the affinity of the SLiM‐based interactions (Kliche & Ivarsson, 2022). Phosphorylation may further affect interactions by conferring structural changes that lead to the exposure or masking of SLiM‐binding sites, or by recruiting competing binding partners. Numerous experimental approaches have been used to explore phospho‐dependent interactions. For example, Tinti *et al* (2013) applied pTyr‐peptide chips in order to probe for the binding preference of SH2 domains. Following alternative two‐hybrid approaches, Grossmann *et al* (2015) performed yeast two‐hybrid experiments with a co‐expressed kinase to capture pTyr‐dependent interactions and Aboualizadeh *et al* (2021) used a mammalian membrane two‐hybrid system to explore the phospho‐dependent interactome of the anaplastic lymphoma kinase (ALK). Moreover, hold‐up assays have been used to screen for phospho‐regulated interactions across the PDZ domain family (Gogl *et al*, 2020). Nonetheless, there is still a paucity of large‐scale methods available for sensitive and specific screening of phospho‐modulated SLiM‐based interactions.

Exploring phospho‐modulated SLiM‐based interactions poses a dual challenge, first to identify SLiM‐based interactions and second to establish the effect of phosphorylation on binding. To address the first challenge, we developed proteomic peptide‐phage display (ProP‐PD; Ivarsson *et al*, 2014; Davey *et al*, 2017; Benz *et al*, 2022). In ProP‐PD, we computationally design a peptide library that tiles the disordered regions of a target proteome and display those peptides on the coat protein of the filamentous M13 phage. The phage peptidome is then used in selections against bait proteins (domains), and the binding‐enriched phage pools are analysed by next‐generation sequencing (NGS) to identify peptides specifically binding to the bait domain (Ali *et al*, 2020; Davey *et al*, 2022). The approach efficiently captures SLiM‐based interactions on a proteome‐wide scale (Benz *et al*, 2022). To simultaneously identify SLiM‐based interactions and obtain an indication of the potential effect of phosphorylation, we introduced in a small‐scale study, focussed on the C‐terminal regions of the proteome, phosphomimetic ProP‐PD (Sundell *et al*, 2018). Here, a phosphomimetic glutamate is used to mimic the effect of serine/threonine phosphorylation on binding. We showed that the glutamate mutations can be used to elucidate the effect of serine/threonine phosphorylation on PDZ domain binding.

In the present study, we sought to generate a ProP‐PD resource, which would allow screening for potentially phospho‐modulated interactions with a broad panel of bait domains. We created a phosphomimetic ProP‐PD library that tiles ~13,500 serine/threonine phosphosites with a design that combines the intrinsically disordered regions of the human proteome (Benz *et al*, 2022) with phosphosites functionally prioritised based on proteomic, structural, evolutionary and regulatory evidence (Ochoa *et al*, 2020). We evaluated the performance of the approach to capture the potential phospho‐modulation of SLiM‐based interactions of 71 domains with a variety of distinct binding preferences. The study represents a major advancement in terms of scale, library design, the diversity of the domain collection screened and in terms of data analysis. Importantly, it shows the general applicability of the approach. Among the novel phospho‐modulated interactions, we highlight the interaction between the clathrin N‐terminal domain (NTD) and the mitotic spindle protein Hepatoma‐upregulated protein (HURP). The interaction was demonstrated to be dependent on S839 phosphorylation in HURP and required for its mitotic function. We show that phosphomimetic ProP‐PD can be used for large‐scale screening of potentially phospho‐modulated SLiM‐based interactions of diverse protein domains. The results can guide further studies on the functional effects of biologically relevant phosphosites within the human proteome. The results are made available in a web‐based resource (http://slim.icr.ac.uk/proppd/), together with other published ProP‐PD results.

## Results

### Design and quality of the internal phosphomimetic ProP‐PD library

**Figure 1:**
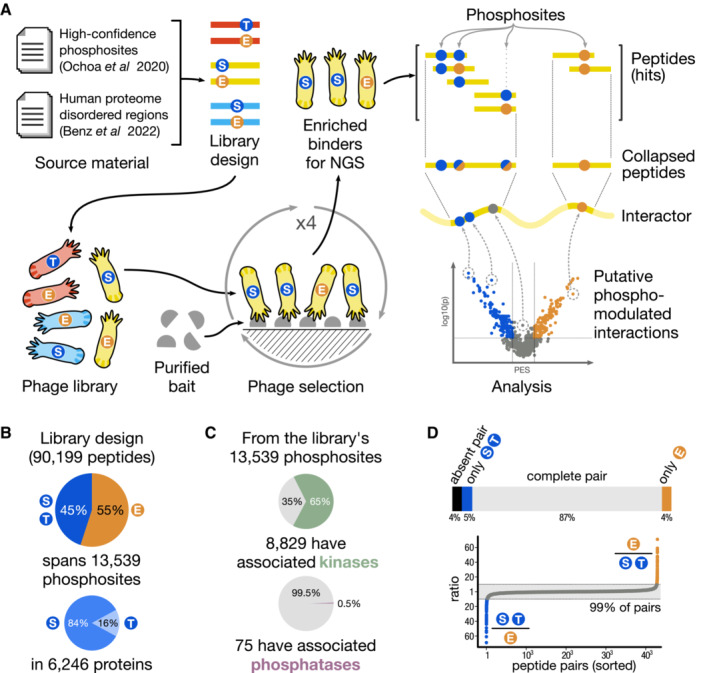
Overview of the workflow and details of the PM_HD2 library Schematic overview of the workflow from library design and phage library construction, to data analysis and validations.Parameters of the PM_HD2 library design.Known associated kinases and phosphatases for the phosphosites included in the PM_HD2 library design.Completeness and proportions of the wild‐type/phosphomimetic peptide pairs in the PM_HD2 library sequencing results.

We designed a novel phosphomimetic human disorderome library (PM_HD2; Datasets EV1 and EV2, and Fig 1A–C) by combining our recently described second‐generation human disorderome (HD2) library (Benz *et al*, 2022) with information on functionally prioritised phospho‐serine/threonine sites (Ochoa *et al*, 2020). The HD2 library tiles the disordered regions of the proteome using 16 amino acid peptides with 12 amino acids overlap between flanking peptides (Benz *et al*, 2022). The functional phosphosite score prioritises phosphosites with a high probability to modulate PPIs, as validated by Ochoa *et al* (2020). The PM_HD2 design contains both the wild‐type peptides with known serine/threonine phosphosites, and serine/threonine to phosphomimetic glutamate mutants thereof. This allows screening of the binding preferences of a given bait protein domain to both the wild‐type and phosphomimetic peptide variants in a single experiment. A custom oligonucleotide library encoding the designed peptide sequences was obtained and was used to fuse the designed peptide sequences N‐terminally to the major coat protein p8 of the M13 bacteriophage. NGS analysis of the constructed PM_HD2 library confirmed at least 91% of the designed sequences were present in the phage library, without any major sequence bias (Fig 1D and Appendix Fig S1D and E).

### The bait domain collection

**Figure 2:**
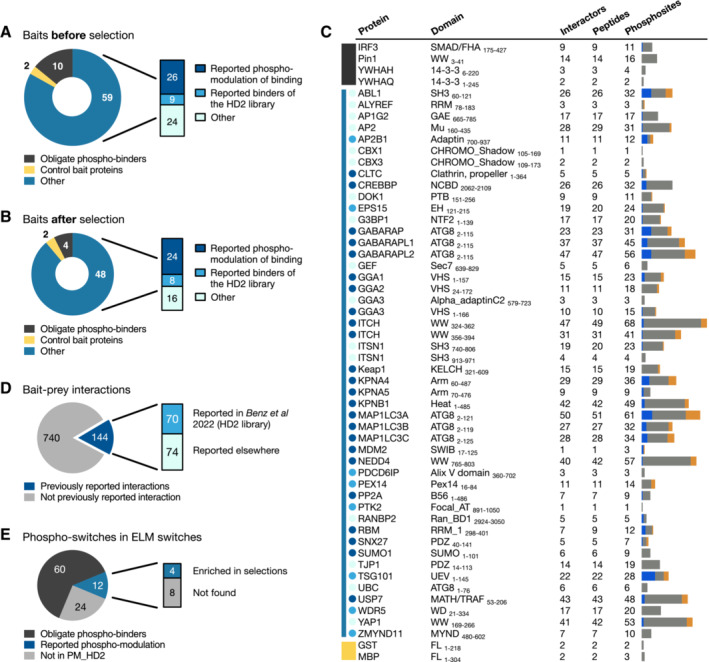
Bait collection and ProP‐PD selection results Overview of the bait protein collection screened against the PM_HD2 library.Bait domains which returned medium/high‐confidence peptides in the screen against the PM_HD2 library.Selection results for each successful bait protein, including the numbers of found interacting proteins, collapsed peptides and phosphosites in the peptides. The colours to the left of the gene names match those of panels (A and B). The bars to the right show the number and distribution of phosphosites, for which we found evidence (*P* ≤ 0.01) of the bait selection favouring the wild‐type (blue) or phosphomimetic (orange) variants. Grey indicates the number of phosphosites for which no preference of wild‐type/mutant variants was observed.Percentage of interactions found that are supported by previous evidence by screening against the HD2 library or reported in HuRi and BioPlex databases (Dataset EV6B).Percentage of phospho‐switches reported in switches. ELM (Van Roey *et al*, 2013) for the bait domain collection and the ones retrieved in selections.

To evaluate the ability of phosphomimetic ProP‐PD to find phospho‐modulated interactions, we designed a bait protein domain collection to cover well‐known obligate phospho‐peptide‐binding domains (i.e. 14‐3‐3 proteins, PLK1 polo‐box domain, IRF3 FHA domain and PIN1 WW domain), peptide‐binding domains for which phospho‐modulation of binding was previously reported (26 domains), well‐known peptide‐binding domains used in our previous selections against the HD2 library (nine domains; Benz *et al*, 2022) and additional peptide‐binding domains to increase the diversity of the peptide‐binding preferences (24 domains; Fig 2A, and Datasets EV3 and EV4). The bait protein domains were glutathione transferase (GST)‐ or maltose binding protein (MBP)‐tagged, and GST and MBP were used as negative controls (Fig 2A and B). The domain collection encompassed 71 bait protein domains covering 38 different domain types (Dataset EV3). The bait protein domains were used in four rounds of selections against the PM_HD2 library. Selections were successful for 54 out of 71 bait protein domains tested based on phage pool ELISA analysis of the binding‐enriched phage pools. Notably, the majority of the selections against obligate phospho‐serine/threonine‐binding domains failed (six out of 10 failed; Fig 2B), whereas the domains with previously reported effect of phosphorylation on binding (24 out of 26 successful) and the remaining peptide‐binding domains (24 out of 33 successful, including reported HD2 binders and others) enriched for one or more medium/high‐confidence peptide during the selections (Fig 2C).

### Phage selection outcome and interaction analysis

The peptide‐encoding regions of the enriched phage pools were barcoded and sequenced by NGS. The NGS results were analysed using a bioinformatic pipeline, where the sequences are translated to peptides, and then matched to the proteome (Benz *et al*, 2022). Peptides were assigned confidence levels (confidence levels: 0 = no confidence, 1 = low, 2–3 = medium and 4 = high confidence) based on an established set of criteria, including occurrence in replicate selections, identification of overlapping peptides and the presence of consensus motifs. For a stringent analysis, we here focus on medium/high‐confidence peptide ligands (Dataset EV5; http://slim.icr.ac.uk/proppd/).

In total, we found 895 domain–peptide interactions involving 54 bait protein domains and 537 peptide‐containing proteins (interactors), which corresponds to 884 identified PPIs. We evaluated whether the selection enriched for interactors of potential biological relevance through a gene ontology (GO) enrichment analysis, using the GO term enrichment analysis tool implemented in PepTools (Benz *et al*, 2022). We found that the selection enriched for peptides from proteins that shared GO terms related to cellular localisation and processes with the bait proteins, in comparison with the background (Appendix Fig S1F and Dataset EV7A). Moreover, 144 (16%) of the interactions found are supported by previous studies, of which approximately 50% were found by previous screening with the HD2 library (Benz *et al*, 2022) and the remainder were found by other methods (Fig 2D and Dataset EV6B).

The identified binding peptides contained 708 unique phosphosites, resulting in a total of 1,105 unique identified bait‐phosphosite pairs since several peptides were found as ligand for more than one bait protein domain (Datasets EV5 and EV6). We evaluated whether the selection returned previously known phospho‐switches by comparing with the instances annotated in the manually annotated switches. ELM resource (Van Roey *et al*, 2013). The database contains 96 previously reported phospho‐switches for our bait protein domain collection of which 72 were part of the PM_HD2 design (Fig 2E and Dataset EV7B); however, most cases (60 phospho‐switches) relate to the obligate phospho‐binders for which the selections returned no peptides or low‐confidence peptides. Of the remaining 12 phosphosites reported to modulate interactions, four were found in our experiments (Fig 2E), including the p53 T18 phosphorylation that negatively regulates the interaction between the MDM2 SWIB domain and p53 (Teufel *et al*, 2009). Consistent with the literature, we found that the phosphomimetic threonine to glutamate substitution negatively affected the p53‐MDM2 interaction (Dataset EV6).

The results for obligate phospho‐binders contained no or few high‐confidence ligands, suggesting that the approach failed to identify ligands for these proteins (Fig 2B and C). Nevertheless, we tested the binding of two of the obligate phospho‐serine/threonine‐binding domains (PIN1 WW domain and 14‐3‐3 theta) to putative binding peptides for which the display indicated a preference for the phosphomimetic peptide (Dataset EV6A). We used wild‐type, phosphomimetic and phosphorylated versions of the peptides in affinity measurements by fluorescence polarisation (FP) displacement experiment. The PIN1 WW domain bound the FITC‐labelled probe peptide (Dataset EV9) but none of the peptides tested (wild‐type, phosphomimetic or phosphorylated S499 PIAS2_493–507_; Appendix Fig S2). Similarly, 14‐3‐3 theta showed no binding for the wild‐type (S375 TOM1_366–382_) and phosphomimetic peptide (S375E TOM1_366–382_), and weak affinity for the phosphorylated peptide tested (pS375 TOM1_366–382_). In conclusion, we found that phosphomimetic ProP‐PD does not capture ligands of obligate phospho‐binding domains, possibly due to a strict requirement of a phosphate moiety for binding to the domains tested. The remainder of our study is thus focussed on cases in which phosphomimetic mutations and phosphorylation abolished or modulated the affinity of interactions.

### Assessment of the identification of putative phospho‐modulated SLiM‐based interactions by ProP‐PD

**Figure 3:**
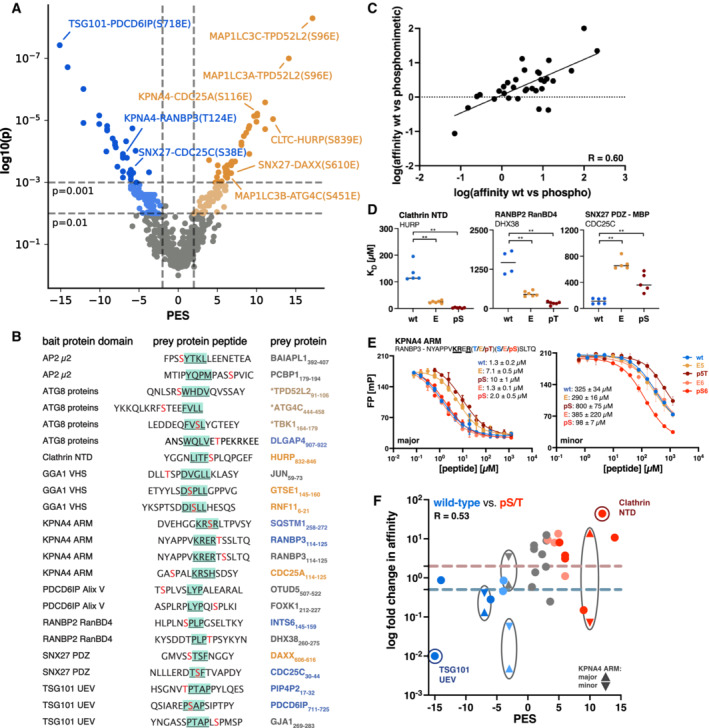
Phosphomimetic mutation in phage display serves as a proxy for preferences for wild‐type or phosphorylated peptides Scatter plot of the PM_HD2 phage selection results indicating pairs with a negative phosphomimetic enrichment (PES) score (left arm; preference for wild‐type peptides) and positive PES (right arm; preference for the phosphomimetic peptides). Coloured peptide pairs represent cases in which the binding preference was statistically significant Mann–Whitney test *P* ≤ 0.01 or *P* ≤ 0.001 and an absolute PES value ≥ 2 with blue pinpointing negative and orange pinpointing positive impact of the phosphomimetic mutation on the binding.Protein domains and peptides used in affinity measurements. Within the peptides, binding motifs are underscored and highlighted in green, and the phosphosites are shown in red. Coloured gene names of the peptide‐containing proteins indicate the suggested phospho‐modulation of the interactions based on ProP‐PD data (blue: disabling and orange: enabling interactions, as in panel A). For KPNA4, affinities were determined for both the major and the minor grove. * = the ATG8 proteins have their individual PES value for those peptides.Correlation of the fold changes in affinity (K_D_ values) between wild‐type/phosphomimetic and wild‐type/phosphorylated peptide pairs (Spearman correlation *R*‐value = 0.60).K_D_ values of wild‐type, mutant and phosphorylated peptides binding to clathrin NTD (HURP_832–846_), RANBP2 RanBD4 (DHX38_260–275_) and SNX27 PDZ (CDC25C_30–44_). Significant differences indicated by the Mann–Whitney test (**: *P* ≤ 0.01). Measurements are from two independent protein preparations with two or three technical replicates each.Displacement curves of the KPNA4 ARM domain with the RanBP3_114–129_ peptides (wild‐type, phosphomimetic and phosphorylated) sampling the effects of two distinct phosphosites (T124 and S125). IC50 values for the different peptides are indicated in μM and with SEM. Measurements were obtained in technical triplicates.Correlation between the fold change in affinity (wild‐type vs. phospho‐peptide) and the PES of the representative peptide pairs based on the PM_HD2 selection data (Spearman correlation *R*‐value = 0.53). Red indicates positive and blue indicates negative phospho‐modulation of the interaction based on the phage selection results. The dotted lines represent the log 2‐fold change in affinity cut‐offs for enabling and disabling interactions, respectively.

In order to deduce information on statistically significant mutation‐modulated interactions from the phosphomimetic ProP‐PD data, we analysed the normalised NGS sequencing counts to calculate a phosphomimetic enrichment score (PES; see Materials and Methods) for each wild‐type and phosphomimetic pair (Dataset EV6). This allowed us to score the observed effects of the phosphomimetic mutations in the ProP‐PD data on the level of the different phosphosites by combining data from multiple peptides and replicates. In order to attribute statistical power to the PES as our scoring metric, we calculate the *P*‐value for each of the interactions based on the Mann–Whitney Test (Fig 3A and Dataset EV6A). By plotting the *P*‐values against the calculated PES, a scatter plot was obtained in which the left arm (blue) indicates interactions negatively affected by phosphomimetic mutations and the right arm (orange) indicates interactions enabled, or promoted, by the phosphomimetic mutations (Fig 3A). For the 1,105 enriched bait‐phosphosite pairs found, 248 (22%) of the phosphosites were suggested to modulate the affinity of the interactions (*P* ≤ 0.01 and absolute PES ≥ 2), and the number decreased to 86 (8%) if applying a more stringent cut‐off (*P* ≤ 0.001). About half of the phosphomimetic mutations had a disabling effect on the interactions, and half had an enabling effect (Dataset EV6A).

We next assessed to what extent the phosphomimetic ProP‐PD correctly captured phospho‐modulated interactions and addressed the two core questions: (i) is the phosphomimetic glutamate mutation a valid proxy for phospho‐serine/threonine, and (ii) does the PES calculated based on the phage display results capture the affinity differences between wild‐type and phosphomimetic/phospho‐peptides. We chose 32 putatively phospho‐modulated interactions covering a window of the PES from −15 to 14 (Fig 3A) and determined affinities of the corresponding bait protein domains for wild‐type, phosphomimetic and phosphorylated variants of the peptides (Fig 3B) through a FP displacement assay (Appendix Figs S3 and S4; Table 1, Dataset EV8 and EV9). The affinities were found to be in the low micromolar to millimolar range (Table 1). The comparison between the effect of phosphorylation and phosphomimetic mutation on binding (assessed by the fold change in affinity for 32 peptide triplets) revealed that there was a clear correlation, with phosphorylation conferring a more pronounced change in affinity (twofold difference) than the phosphomimetic mutations (Fig 3C). This observation is likely explained by the net charge difference between a phospho‐serine/threonine and a glutamate. The interaction of the PDZ domain of SNX27 with ligands with an internal PDZ binding motif (x(T/S)xFx, with F = p0; found in DAXX_606–616_ and CDC25C_30–44_) stood out as an exception to the general rule. For these interactions, we found that the two phosphomimetic mutations tested (p − 1 in CDC25C_30–44_ and p − 3 in DAXX_606–616_; Fig 3B) caused a higher fold change in affinity in comparison with the phosphorylated peptides (Table 1). In order to strengthen our affinity data set, we repeated a selected subset of FP‐based affinity measurements using independently prepared protein domains (Fig 3D). The FP repeat measurements confirmed significant differences in affinity for the wild‐type peptides as compared to phosphomimetic and phosphorylated peptides for clathrin NTD for HURP_832–846_ (enabling effect), RANBP2 RanBD4 for DHX38_260–275_ (enabling effect) and SNX27 PDZ for CDC25C_30–44_ (disabling effect), consistent with the phosphomimetic ProP‐PD results. The affinity results were further complemented by isothermal titration calorimetry (ITC) measurements for clathrin NTD, GGA1 VHS and MAP1LC3A and ‐B (Appendix Figs S5A–D and Dataset EV10). The affinities observed by ITC were in good agreement with the FP measurements (Table 1 and Dataset EV10).

**Table 1:** Overview of affinity data.

Protein bait domain	Phosphosite relative to SLiM	K_D_ values ± SEM (μM)			PES (*P*‐value)
		Wild‐type	E	pS	
AP2 μ2	p − 1 (BAIAPL1_392–407_)	1.29 ± 0.06	0.49 ± 0.08	0.93 ± 0.01	1.2 (0.095)
	p + 8 (PCBP1_179–194_)	3.7 ± 0.1	2.05 ± 0.09	0.67 ± 0.04	1 (0.15)
Clathrin NTD	p + 5 (HURP_832–846_)	134 ± 16	23 ± 2	pS: < 3 pS Cterm: < 3^a^	12 (0.000009)
GABARAPL2	p − 4 (ATG4C_444–458_)	0.8 ± 0.4	1.8 ± 0.2	0.1 ± 0.2	4.3 (0.0025)
	p + 3 (TBK1_164–179_)	19 ± 4	12.4 ± 0.6	2.8 ± 0.6	1 (0.11)
	p + 6 (DLGAP4_907–922_)	174 ± 22	260 ± 25	150 ± 32	−3.9 (0.00047)
GGA1 VHS	p − 3 (JUN_59–73_)	470 ± 50	450 ± 50	1,900 ± 700	3.6 (0.023)
	p + 1 (GTSE1_145–160_)	96 ± 8	199 ± 4	640 ± 70	9 (0.00012)
	p + 2 (RNF11_6–21_)	420 ± 50	1,000 ± 230	31 ± 3	4.9 (0.009)
KPNA4 ARM Major	p − 4 (CDC25A_114–125_)	8.8 ± 0.2	0.75 ± 0.03	0.63 ± 0.04	10 (0.000006)
	p + 3 (SQSTM1_258–272_)	0.24 ± 0.04	5.3 ± 0.4	50 ± 7	−3 (0.0096)
	p + 5 (RANBP3_114–129_)	0.15 ± 0.01	0.80 ± 0.03	1.13 ± 0.05	−7 (0.00012)
	p + 6 (RANBP3_114–129_)	0.15 ± 0.01	0.14 ± 0.01	0.22 ± 0.03	−3.1 (0.077)
KPNA4 ARM minor	p − 4 (CDC25A_114–127_)	0.200 ± 0.004	2.3 ± 0.3	2.8 ± 0.4	10 (0.000006)
	p + 3 (SQSTM1_258–272_)	14.0 ± 0.7	41 ± 2	248 ± 3	−3 (0.0096)
	p + 5 (RANBP3_114–129_)	68 ± 4	61 ± 2	167 ± 9	−7 (0.00012)
	p + 6 (RANBP3_114–129_)	68 ± 4	80 ± 27	20 ± 1	−3.1 (0.077)
MAP1LC3A	p − 4 (ATG4C_444–458_)	5.2 ± 0.2	3.1 ± 0.2	1.3 ± 0.1	2.3 (0.015)
	p − 1 (TPD52L2_91–106_)	226 ± 14	78 ± 3	20.6 ± 0.6	14 (0.000000099)
	p + 3 (TBK1_164–179_)	8.4 ± 0.3	4.8 ± 0.4	0.89 ± 0.05	3.2 (0.0061)
MAP1LC3B	p − 4 (ATG4C_444–458_)	7.1 ± 0.6	4.6 ± 0.5	1.9 ± 0.3	6 (0.00063)
	p − 1 (TPD52L2_91–106_)	760 ± 150	225 ± 17	61 ± 2	3 (0.012)
	p + 3 (TBK1_164–179_)	10.5 ± 0.1	2.1 ± 0.2	1.3 ± 0.3	5.1 (0.00047)
PDCD6IP Alix V domain	p − 5 (OTUD5_507–522_)	68 ± 6	88 ± 7	116 ± 20	0.66 (0.34)
	p + 6 (FOXK1_212–227_)	25 ± 3	13 ± 1	13 ± 2	2.9 (0.04)
RAN2BP RanBD4	p − 1 (INTS6_145–159_)	334 ± 35	1,075 ± 85	733 ± 128	−4 (0.0044)
	p + 4 (DHX38_260–275_)	1,500 ± 190	470 ± 35	167 ± 21	3 (0.04)
SNX27 PDZ	p − 3 (DAXX_606–616_)	300 ± 125	23 ± 4	96 ± 5	6 (0.00063)
	p − 1 (CDC25C_30–44_)	111 ± 17	680 ± 40	395 ± 63	−6 (0.00063)
TSG101 UEV	p − 1 (PIP4P2_17–32_)	14 ± 1	18 ± 2	16 ± 3	−14 (0.0000002)
	p + 5 (GJA1_269–283_)	232 ± 16	115 ± 4	209 ± 8	6 (0.0013)
	p + 2 (PDCD6IP_711–725_)	16 ± 3	n.b.	n.b.	−15 (0.00000004)

The protein bait domain, the phosphosite position relative to the SLiM and the K_D_ values of wild‐type, phosphomimetic and phosphorylated peptides with SEM are indicated (at least three technical replicates). For comparison, the calculated PES score is indicated.

^a^ The affinity for the phospho‐HURP peptides was estimated due to the peptides binding with considerably higher affinity than the FITC‐peptide which limits the calculation capacity of K_D_.

The binding of the KPNA4 ARM domain is complex in that the protein has two binding pockets (major and minor) that bind basic motifs of different classes (Benz *et al*, 2022). Using probe peptides for the distinct binding pockets (Fig 3E), we observed that the affinities of the peptides were generally higher for the major pocket, suggesting that this was the main pocket probed during the phage selection. We further found that the effects of the modifications were different depending on the binding pocket. Notably, we found that the PES reflected the change in affinity for the major pocket. This becomes particularly apparent for the CDC25A_114–125_ peptides, for which the two binding pockets show opposite preferences for pS116 (Table 1). We thus used the results for the KPNA4 major pocket for the following evaluation.

We tested 28 bait‐peptide triplets, on the phosphosite level, to evaluate if the calculated PES (absolute PES ≥ 2) and the associated *P*‐value (*P* ≤ 0.01) correlated with the fold change in affinity elicited by phosphorylation. For 14 of the 18 (78%) tested putatively phospho‐modulated interactions that complied to the cut‐off values, there was a good agreement between the PES and the fold change in affinity (at least twofold) between wild‐type/phosphorylated peptides in terms of the effect on binding (Fig 3F). Increasing the stringency further (*P* ≤ 0.001) conferred a minor improvement in the accuracy of identification of phospho‐modulated interactions (nine out of 11, 82% agreement). For 10 of the interactions tested, the associated *P*‐value was > 0.01, and for most of them, phosphorylation had either no effect on binding or had an effect with poor correlation with the PES. This argues in favour of the chosen cut‐off values. The combined score performed well both in indicating disabling (5 out of 7 confirmed) and enabling (9 out of 11 confirmed) effects on binding. For 22% (4 out of 18) of the cases, the PES failed to correctly predict the effect of phosphorylation on binding. Of these cases, two were TSG101 UEV ligands, where phosphorylation on the positions flanking the P(S/T)AP motif (p − 1 and p + 5) had neutral effects, but were indicated as having either enabling or disabling effects by the phosphomimetic ProP‐PD data. In contrast, the analysis correctly identified the effect of phosphorylation at the p + 1 position on the core motif residue. The results suggest that the data may be noisy for positions that are in motif‐flanking positions when the overall effects on binding are neutral. A similar result was observed for GABARAPL2 and the p + 6 position. For the last case, the binding of GGA1 VHS domain to GTSE1_145–160_, the affinities for all peptides were low, which may have rendered the results of the affinity measurements less accurate.

Overall, the analysis supports that phosphomimetic ProP‐PD is a useful approach to identify SLiM‐based interactions and simultaneous probe the effects of phospho‐modulation, in particular when the phosphorylation occurs within the core consensus motif.

### Position‐wise analysis of the effect of phosphomimetic mutation and phosphorylation in relation to consensus motifs

**Figure 4:**
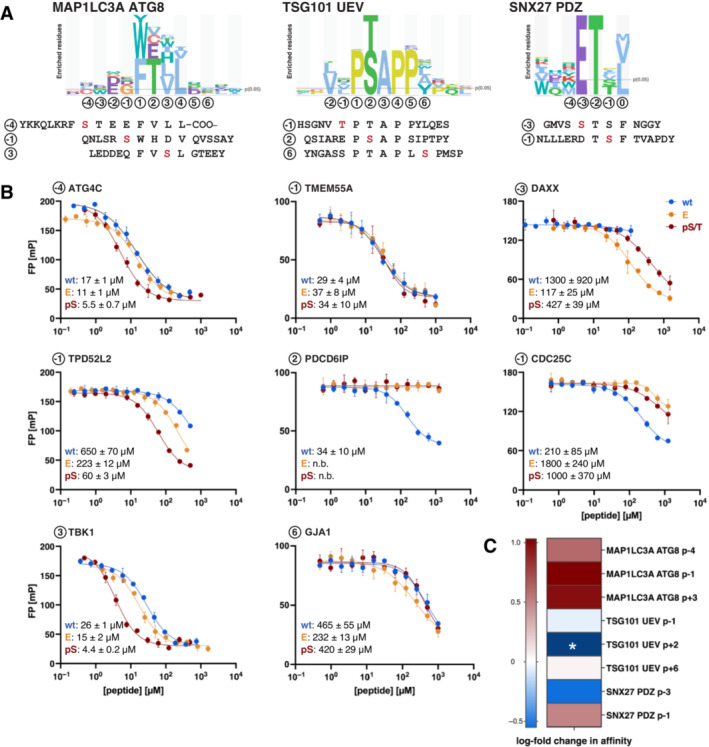
Preferences in binding motifs for serine/threonine or aspartate/glutamate are, respectively, indicative for negative or positive phospho‐modulation Sequence logos of peptides binding to MAP1LC3A ATG8 and TSG101 UEV domain found through previous selections against the HD2 library (Benz *et al*, 2022), together with the C‐terminal PDZ binding motif of the SNX27 PDZ domain. Peptides and phosphosites tested by affinity measurements are indicated and aligned to the motifs.Displacement curves of the three different bait domains and the indicated peptides using wild‐type, phosphomimetic and phosphorylated peptide. IC50 values of the different peptides are indicated in μM and with SEM. Measurements were in technical triplicates.Fold changes in affinity (wild‐type vs. phospho‐peptides) with blue indicating negative and red indicating positive phospho‐modulation of the tested interactions. *The exact fold change for the TSG101 UEV domain–PDCD6IP peptide interaction could not be calculated as the fitting of the displacement curve of the phosphorylated peptide did not provide a IC50 value due to the weak affinity.

Phosphomimetic ProP‐PD offers the possibility to simultaneously identify SliM‐based interactions and to gain position‐wise information on the effect of phosphosites in relation to the consensus motif of a given protein domain. A relevant question is whether it is possible to predict the effect of phosphorylation based on the results and motifs generated using a conventional ProP‐PD library displaying only wild‐type sequences or using a combinatorial peptide‐phage library, as previously suggested (Benz *et al*, 2022). We hypothesise that the binding motifs of the individual domains have predictive value when it comes to the position‐wise effect of phosphorylation. Acidic residues may serve as positive indicators and serine/threonine residues as negative indicators. We explored this using the MAP1LC3A ATG8, the TSG101 UEV and the SNX27 PDZ domains as model baits. MAP1LC3A is known to bind a LC3‐interacting region (LIR) motif, which centres around an aromatic residue at p + 1 position and a hydrophobic residue at p + 4, interspaced by two variable amino acids ([[F/Y/W]xxɸ]; Pankiv *et al*, 2007; Johansen & Lamark, 2020; Fig 4A). We tested the phospho‐modulation of the MAP1LC3A interactions by using LIR‐containing peptides phosphorylation at position p − 4, p − 1 and p + 3. The protein bound all tested phospho‐peptides with higher affinity than the unphosphorylated peptides, which is in agreement with the results of the phosphomimetic ProP‐PD (Fig 4B and C, Table 1). A comparison with the residues favoured at these positions as determined by previous conventional ProP‐PD selections against the HD2 library (Benz *et al*, 2022, Fig 4A), revealed a partial enrichment of acidic residues (aspartate/glutamate) at the p − 4 and p − 1 positions consistent with the observed effects of phosphorylation. In contrast, the affinity‐enhancing effect on MAP1LC3A binding observed for phosphorylation of the p + 3 position was not revealed by the consensus motif, but by the phosphomimetic phage display developed here.

The TSG101 UEV domain binds to a P[S/T]AP motif (Pornillos *et al*, 2002) in which the second position (p + 2) occupied by a serine/threonine makes part of the consensus previously generated by phage display (Teyra *et al*, 2020; Benz *et al*, 2022). Based on the motif, it is thus likely that phosphorylation of the p + 2 position would impact binding negatively. Using wild‐type, phosphomimetic and phosphorylated PDCD6IP_711–725_, we confirmed that both phosphomimetic mutation and threonine phosphorylation at the p + 2 position abolished binding (Fig 4B and C). In contrast, modifications of the flanking residues at p − 1 or p + 5 positions of the PIP4P2 and GJA1 peptides had no effect on binding as discussed above. In line with these results, the TSG101 consensus binding motif has no acidic (aspartate/glutamate) or serine/threonine residues enriched at those positions (Fig 4A). For the SNX27 PDZ domain, which binds to a class I PDZ binding motif (S/T‐x‐ɸ‐COOH with the last position being p0; Gallon *et al*, 2014), we found that the affinity was enhanced by phosphorylation at position p − 1 and negatively affected by p − 3 phosphorylation (Fig 4B and C), as indicated by the phosphomimetic ProP‐PD and reflected by a strong preference for either acidic residues (p − 3) or serine/threonine (p − 1) at the respective positions revealed by previous phage display selection data (Fig 4A).

To summarise, we find that it is to some extent possible to postulate the effects of serine/threonine phosphorylation based on consensus motifs generated from phage display selections against a wild‐type ProP‐PD library (or a combinatorial library) but that the phosphomimetic approach provides additional information.

### Linking readers to writers and erasers on a large scale

**Figure 5:**
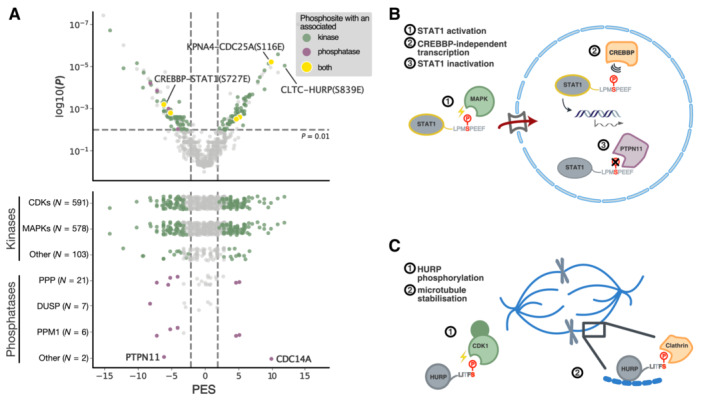
Annotated kinases and phosphatase for sites in the medium/high‐confidence ligands Top: Phosphosites in medium/high‐confidence ligands associated with kinases (green) and phosphatases (purple). Yellow indicates cases in which both kinase and phosphatase are known. Bottom: Associated kinases and phosphatases of medium/high‐confidence ligands across different prioritisation of their effect on binding (grey—insignificant effects, coloured—significant as judged by the PES and a *P*‐value ≤ 0.01 of the Mann–Whitney test).Phosphorylation of STAT1 by MAPKs at S727 results in its activation as transcriptional regulator in response to inflammatory stimuli (Kovarik *et al*, 1999; Varinou *et al*, 2003). The interaction of STAT1 with CREBBP NCBD is inhibited (Wojciak *et al*, 2009), which points at CREBBP‐independent transcription upon S727 phosphorylation. Dephosphorylation of S727 is mediated by PTPN11 (Wu *et al*, 2002).Phosphorylation of HURP at S839 by CDK1 enables potentially its interaction with clathrin and together they may contribute to the microtubule stability during mitosis.

Of the 248 significantly phospho‐modulated interactions discovered, 155 (62%) involved phosphosites associated with a known kinase with the vast majority (85%) being targets of mitogen‐activated protein kinases (MAPKs) and/or cyclin‐dependent kinases (CDKs). This is similar to the distribution in the original library (78% of sites associated with MAPKs and/or CDKs; Appendix Fig S1C). For most of the phosphosites, several kinases are predicted, in which the [ST]P‐modifying proline‐directed kinases MAPK and CDK targets largely overlap (MAPKs and CDKs: 126; CDKs only: 6; other: 23; Fig 5A and Dataset EV6A). In turn, nine out of the 248 phosphosites have an annotated phosphatase (3.6%), which represents an increase in percentage in comparison with 0.5% of the peptides in the library design (Fig 1C). For four cases, we found putative phospho‐switches with information on both the kinases and phosphatases that modify the position. Among them, we note the interaction between KPNA4 ARM and the CDC25A_114–125_ peptide where S116 phosphorylation causes a switch in the pocket preference of KPNA4 ARM domain (Fig 3E and Table 1), and the binding of the CREBBP NCBD domain to the STAT1_719–735_ peptide (Fig 5A and B) which is inhibited by the phosphomimetic S727E mutation. However, for most cases, there is only information on the predicted kinases. For example, we identified a phospho‐modulated interaction between the N‐terminal domain (NTD) of clathrin and the C‐terminal peptide of Hepatoma‐upregulated protein (HURP), which was suggested by the phage display to be positively modulated by phosphorylation at S839 (Dataset EV6A) by a proline‐directed kinase (MAPKs or CDK1; Fig 5A and C).

### Phosphomimetic ProP‐PD reveals a phospho‐modulated clathrin‐binding motif in HURP that is necessary for normal mitotic progression

**Figure 6:**
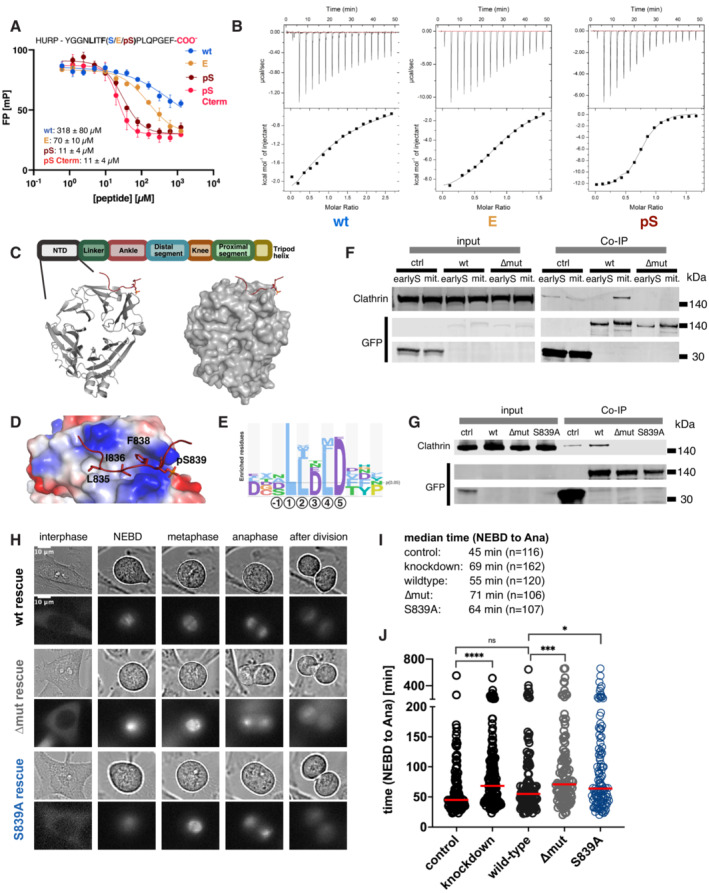
Clathrin NTD binds to a phospho‐modulated motif in the HURP C terminus, which is required for HURP mitotic function ADisplacement curves of the FP experiment using clathrin NTD and wild‐type, phosphomimetic and phosphorylated HURP peptides. IC50 values are indicated in μM and with SEM. Measurements were in two to three technical replicates per protein preparation (2 independent preparations).BITC experiments of the clathrin NTD and wild‐type, phosphomimetic and phosphorylated HURP peptides. Measurements were in at least technical triplicates.CSchematic overview of the domain organisation of clathrin, together with crystal structure of clathrin NTD in complex with the Ser839 phosphorylated HURP_826‐846_ peptide (PDB: 7ZX4).DElectrostatic representation of the clathrin NTD bound to the Ser839 phosphorylated HURP_826‐846_. The phosphate group is complex by two basic residues R64 and K96.ESequence logo representation of clathrin NTD binding peptides listed in ELM (Kumar *et al*, 2022).FCo‐IP of clathrin with Venus‐tagged HURP_WT_ and HURP_ΔMUT_ constructs in early S phase and mitotic HeLa cells.GCo‐IP of clathrin with Venus‐tagged HURP_WT_, HURP_ΔMUT_ and HURP_S839A_ constructs in mitotic HeLa cells.HRepresentative images of HURP_WT_, HURP_ΔMUT_ and HURP_S839A_ in the different mitotic stages.I, JRNAi rescue experiments (*n* = 2), which included the knockdown of endogenous HURP and re‐expressing either RNAi‐resistant HURP_WT_, HURP_ΔMUT_ and HURP_S839A_. In order to assess mitotic behaviour of the cells, the time required of control, HURP knockdown, HURP_WT_, HURP_ΔMUT_ and HURP_S839A_ HeLa cells to progress through mitosis (NEBD‐Ana) was established using time‐lapse microscopy. Every circle represents one analysed cell. The number of cells analysed is indicated as *n* in (I). Mann–Whitney test was performed to assess significant differences in the median time required for mitotic progression (*P‐value ≤ 0.05, ***P‐value ≤ 0.001, ****P‐value ≤ 0.0001; ns, nonsignificant). Source data are available online for this figure.

We hypothesised that the interaction between HURP and clathrin could be involved in the regulation of microtubule dynamics at the mitotic spindle as both proteins are reported to be involved in mitotic spindle organisation (Wong & Fang, 2006; Royle, 2012; Rondelet *et al*, 2020). Further, HURP is highly phosphorylated during mitosis, suggesting that the proline‐directed phosphorylation on S839 is a CDK target (Hsu *et al*, 2004; Yu *et al*, 2005; Wong & Fang, 2006), which prompted us to investigate the interaction in this cell cycle stage (Fig 5C). We found that phosphorylation of S839 in the HURP_832–846_ peptide results in a 20‐fold increase in affinity, as demonstrated by both FP and ITC experiments (HURP_832–846_: FP: K_D_ ≈ 150 μM and ITC: K_D_ ≈ 250 μM, pS839 HURP: FP: K_D_ < 3 μM and ITC K_D_ = 5 μM; Fig 6A and B, Table 1, and Dataset EV10). This is to our knowledge the first report of an interaction between clathrin and a peptide phosphorylated at the p + 5 position. Previously, it has been shown that phosphorylation exposes a peptide‐binding site for the clathrin ankle domain, an interaction that does not involve direct phosphate group recognition (Burgess *et al*, 2018). Clathrin NTD has four reported binding sites to accommodate its adaptor proteins (Willox & Royle, 2012), and HURP_832–846_ could potentially bind to one or more of them. To shed light on the structural details of the complex, we co‐crystallised a pS839 HURP_826–846_ peptide with the clathrin NTD. The protein crystallised as a dimer and the peptide bound to the binding pocket 1 in both monomers (Fig 6C) but was also found to bind to pocket 3 in one of the monomers. In pocket 1, the phosphate group is clamped between the basic residues R64 and K96 (in 4.2 and 2.7 Å distance, respectively) and hence at an appropriate distance to form salt bridges with the phosphate group (Fig 6D). In comparison, only one basic residue (K227) was found in vicinity of the phosphate group in pocket 3. Both pockets can hence accommodate the LIxF‐motif, although pocket 1 appears to be the preferred binding pocket due to the observed occupancy and due to a better accommodation of the phosphate group. Several clathrin‐binding peptides with acidic residues at the p + 5 position have previously been reported (ter Haar *et al*, 2000; Dell'Angelica, 2001; Rondelet *et al*, 2020), as evidenced by the ligands curated in ELM (Fig 6E) (Kumar *et al*, 2022). Superimposition with previously reported structures of the clathrin NTD with a LIxFD‐containing peptide shows that the acidic residue on the bound peptide assumes a similar, but not identical orientation as the phosphorylated serine in pS839 HURP_826–846_ (Appendix Fig S6A).

To assess the biological relevance of the discovered clathrin‐HURP interaction, we aimed to validate the binding in context of the full‐length proteins in a cellular setting. Since S839 is a putative CDK1 site and HURP is reportedly phosphorylated in mitosis (Hsu *et al*, 2004; Yu *et al*, 2005), we focussed on this cell cycle stage. To this end, we generated HeLa cells stably expressing inducible Venus‐tagged wild‐type HURP (HURP_WT_), C‐terminally truncated HURP (HURP_ΔMUT_) where the last 12 amino acids are deleted, or a  S839A HURP (HURP_S839A_) mutant where the phosphosite essential for clathrin binding was disrupted. We confirmed the interaction between HURP_WT_ and clathrin in mitotic HeLa cells and observed that the interaction was abolished by deletion of the HURP C‐terminal region and by the S839A mutation (Fig 6F and G). In contrast, the co‐immunoprecipitation of HURP_WT_ by clathrin failed when it was performed using early S phase cells, thus supporting that the interaction is restricted to mitosis, when CDK activity is high (Fig 6F). Taken together, this confirmed the requirement of the C‐terminal LIxF‐motif and the S839 phosphosite in HURP for its interaction with clathrin in mitosis. Following these results, we assessed the functionality of the clathrin‐binding motif and the S839 phosphosite for the mitotic function of HURP. It has previously been reported that knockdown of HURP results in a mitotic delay observed as a prolonged time required to progress from nuclear envelope breakdown (NEBD) to anaphase (Ana; Wong & Fang, 2006). Consistently, we observed a delay in mitotic progression when we knocked down endogenous HURP (Fig 6H–J) by RNA interference (RNAi; median time (knockdown) = 69 min, median time (control) = 45 min; Fig 6H–J). To test whether HURP interaction with clathrin is necessary for normal mitotic progression, we subsequently induced the expression of RNAi‐resistant HURP_WT_, HURP_ΔMUT_ and HURP_S839A_. All protein variants displayed the same localisation and associated with the spindle during mitosis. However, while HURP_WT_ efficiently rescued the RNAi of endogenous HURP (median time = 55 min), HURP_ΔMUT_ (median time = 71 min) and HURP_S839A_ (median time = 64 min) did not. In conclusion, the association of HURP and clathrin through the C‐terminal LIxF‐motif and enabled by S839 phosphorylation is required for the mitotic function of HURP.

Related to mitotic function of clathrin, a recent study showed that the G2 and S phase‐expressed protein 1 (GTSE1) contains several clathrin‐box motifs and is recruited to the mitotic spindle through interactions with clathrin. The interaction between the two proteins has a microtubule stabilising function (Rondelet *et al*, 2020). In our phosphomimetic phage display, we also found clathrin NTD to bind to a GTSE1 peptide containing two closely linked LIxF‐motifs (_692−_GQ**LIDL** *SS*P**LIQL** *S*PE_−707_) of which the first has been co‐crystallised in the pocket 3 of clathrin (Rondelet *et al*, 2020). The second motif has been found in the clathrin pocket 1, although it was suggested that this observation may be due to a crystallisation artefact. Nonetheless, overlaying of the crystal structures with complexed HURP or GTSE1 peptides suggests that the two proteins could compete for binding (Appendix Fig S6B). The clathrin‐binding GTSE1_692–707_ peptide we identified in our display contains three reported serine phosphorylation sites, of which one was included in our library design (S705; the other two positions were not probed due to lower functional scores). The phosphomimetic ProP‐PD results suggested that S705 phosphorylation had little effect on clathrin binding (Dataset EV6A). We explored the effect of GTSE1 phosphorylation on positions S698, S699 or S705 on clathrin NTD binding in a displacement experiment. We found the affinity of the GTSE1_692–707_ peptide for clathrin to be ≈ 100 μM, but that there was no difference in the binding affinity between the unphosphorylated and the S698, S699 and S705 phosphorylated peptides (Appendix Fig S6C and D).

In conclusion, our results suggest that GTSE1 and HURP could compete for clathrin binding, but that the interaction with HURP is uniquely phospho‐regulated. Notably, GTSE1 harbours two additional clathrin‐binding motifs (Rondelet *et al*, 2020). The high affinity of the S839 phosphorylated HURP may thus be required to confer competitive advantage to HURP for binding clathrin within a cellular context.

## Discussion

In this study, we established phosphomimetic ProP‐PD as a general approach to identify phospho‐serine/threonine‐modulated motif‐based interactions. We identified 895 peptide‐domain interactions of which 22% are suggested to potentially be modulated by phosphorylation. We validated the results by affinity measurements of 32 wild‐type, phosphomimetic and phospho‐peptide triplets. We found that phosphomimetic serine/threonine to glutamate mutations mimicked the effect of phosphorylation for phospho‐modulated interactions, although in most cases with a less pronounced effect on affinity. Based on our experimental validations, phosphomimetic ProP‐PD correctly identified 78% (*P* ≤ 0.01) of the tested interactions in terms of preferential binding to either wild‐type or phospho‐peptides (82% with higher confidence, *P* ≤ 0.001). This highlights the potential of phosphomimetic ProP‐PD to simultaneously provide large‐scale information on novel interaction partners and on the potential phospho‐modulation of binding. A main advantage of phosphomimetic ProP‐PD is that it permits the identification of interactions that are enabled by the phosphomimetic mutations that would otherwise not be captured using a library of wild‐type sequences. The conservative library design used ensures that the phosphosites tested are of high confidence with respect to their localisation and functionality. This enables the discovery of biologically relevant phosphosites with impact on binding interfaces. For cases where the results suggest reduced affinity upon phosphomimetic mutations of core motif residues, other mutations (e.g. disease‐related mutations) may similarly perturb binding.

By combining the results of the interaction screening with information on associated kinases and phosphatases, we provide novel insights into the system of phospho‐writers, readers, and erasers, which can place the interaction in a certain biological setting and in this fashion facilitate potential follow‐up studies. For example, our results revealed a phospho‐dependency of the clathrin‐HURP peptide interaction. The role of clathrin is well established in endocytosis, but has also been extended to mitosis, during which clathrin is an essential player of microtubule stability (Royle, 2012; Rondelet *et al*, 2020). Clathrin classically interacts with its adaptors via clathrin adaptor motifs, such as the LIxF‐motif (Dell'Angelica, 2001; Willox & Royle, 2012; Benz *et al*, 2022). HURP, in turn, is a microtubules binding protein, the knockdown of which results in a mitotic delay (Wong & Fang, 2006). The predicted kinase for the phosphosite is CDK1, which contextualises the two proteins to a shared biological setting. We showed that this interaction is positively regulated by phosphorylation on a site adjacent to the LIxF‐motif and provided a structural explanation for its phospho‐dependency. We further confirmed the clathrin‐HURP interaction in mitotic HeLa cells and its dependency on the LIxF‐motif and S839 phosphorylation. We moreover showed that both motif and S839 phosphorylation are required for the mitotic function of HURP. This strengthens the notion that phosphomimetic ProP‐PD can delineate interactions with functional relevance in the cell while providing mechanistic details on the amino acid level, including binding motifs and putative phospho‐modulation.

A main limitation of the phosphomimetic ProP‐PD approach is that it does not capture interactions of obligate phospho‐binders. This limitation applies to all studies using phosphomimetics, such as cell‐based studies in which it is frequently used as a proxy for the phosphorylated protein due to the ease of introducing the mutation. Another limitation lies in the constraints we imposed on the design by using the high‐confidence phosphosites suggested by the study by Ochoa *et al* (2020). In particular, the library design is likely skewed towards substrates of well‐known kinases with known motifs, as the kinase motifs were used as one among several features to identify functionally important sites. In the future, it can be envisioned to make an updated library, as the phosphorylation motifs of the full serine/threonine kinome were recently released (Johnson *et al*, 2023). Other approaches will be required to identify the interactions of obligate phospho‐binders, such as for example prephosphorylation of phage libraries using kinases (Dente *et al*, 1997; Sundell *et al*, 2018), yeast two‐hybrid in presence of kinases (Grossmann *et al*, 2015), peptide array‐based approaches, phospho‐peptide pull down coupled to mass spectrometry (Brandt *et al*, 2006), or using an expanded genetic code (Rogerson *et al*, 2015). Furthermore, we only sample 71 domains of thousands of potential SLiM‐binding domains in the human proteome, and thus, we only explore a fraction of the interaction space. The number of phospho‐serine/threonine‐modulated interactions reported in this study likely represents only a small fraction of phospho‐modulated binding sites in the tiled regions.

It should further be noted that phosphorylation of disordered regions may have other functions than modulating SLiM‐based interactions. For example, it may modulate the interactions between intrinsically disordered regions and RNA, which, for example, may regulate the partitioning of the proteins into biomolecular condensates (Sridharan *et al*, 2022). Other functional consequences of phosphorylation in disordered regions may be disrupting interactions with membranes (e.g. as shown for the MARKS peptide), modulating structural dynamics, or induce folding (Bah *et al*, 2015). Of note, we only explore here serine/threonine phosphorylation, while other phosphorylation events have important roles in cell function. Thus, much remains to be done before we have a comprehensive understanding of the functional effects of phosphorylation. The phosphomimetic ProP‐PD approach outlined here will be a useful addition to the field, with the main advantages being the detailed simultaneous information on the SLiM‐binding sites, information on positive or negative modulation of the interaction by phosphorylation and the scalability of the approach.

## Materials and Methods

### Reagents and Tools table

**Table:** 

Reagent/Resource	Reference or Source	Identifier or Catalog Number
**Experimental Models**		
*Escherichia coli* BL21(DE3) gold	Agilent Technology	Cat. 230132
*Escherichia coli* OmniMAX	ThermoFisher	Cat. C854003
*Escherichia coli* SS320	Lucigen	Cat. 60512‐1
M13KO5 helper phage	ThermoFisher	Cat. 18311019
Flp‐In T‐RExHeLa cells	Stephen Taylor	
**Recombinant DNA**		
pETM33	EMBL	
pETM41	EMBL	
Phagemid p8	Sidhu lab (PMID: 25879139)	
HURP cDNA	CPR clone resource	
YFP pcDNA/FRT/TO	Nilsson lab – cloning YFP with HindIII in pcDNA5/FRT/TO	
**Antibodies**		
HRP‐conjugated anti‐M13 bacteriophage monoclonal mouse antibody	Sino Biological Inc	Cat. 11973‐MM05T‐H/RRID: AB_2857928
anti‐CHC polyclonal rabbit antibody	Bethyl Laboratories	Cat. A304‐743A‐M/RRID: AB_2782136
anti‐HURP polyclonal rabbit antibody	Bethyl Laboratories	Cat. A300‐852‐M/RRID: AB_2779502
anti‐GFP (mouse/rabbit) antibody	rabbit‐ anti GFP, produced in‐house or mouse anti‐GFP from Roche	Cat. 11814460001/RRID: AB_390913 for Roche
IRDye® 800CW Goat anti‐Mouse IgG Secondary Antibody	LI‐COR	Cat. 926‐32210/RRID: AB_621842
IRDye® 680RD Goat anti‐Rabbit IgG Secondary Antibody	LI‐COR	Cat. 926‐68071/RRID:vAB_10956166
**Oligonucleotides and sequence‐based reagents**		
RNA oligos/Ambion™ Silencer™ Select Pre‐Designed siRNA	Thermo Fisher	s18913
Oligonucleotides PM_HD2	CustomArrray	
HURP_fwd_in_YFP	GATCGGATCCATGTCTTCATCACATTTTGCC	
HURP_rev_in_YFP_wt	GATCGCGGCCGCTCAAAATTCTCCTGGTTGTAGAGG	
HURP_rev_in_YFP_delta835‐846	GATCGCGGCCGCTCAGTTACCACCAAAAGAAATGTGTC	
HURP_fwd_S839A	GGTAACCTGATTACTTTTGCACCTCTACAACCAGGAG	
HURP_rev_S839A	CTCCTGGTTGTAGAGGTGCAAAAGTAATCAGGTTACC	
SiRes_HURP_fwd_483	GATAACGAGAGTGACGTGAGGGCGATCCGACCTGGTCC	
SiRes_HURP_rev_483	GGACCAGGTCGGATCGCCCTCACGTCACTCTCGTTATC	
SiRes_HURP_fwd_484	ATGCCGGTCCTCAAAACACAAAGAGTGAACATGTGAAG	
Sires_HURP_rev_484	CTTCACATGTTCACTCTTTGTGTTTTGAGGACCGGCAT	
**Chemicals, enzymes and other reagents**		
Zeocin	Invitrogen	Cat. R25001
Blasticidin	Invitrogen	Cat. R21001
Doxycyclin	Clontech Labs	631311 – 5g
Nocodazole	Sigma	Cat. M1404
Lipofectamine™ RNAiMAX Transfection Reagent	Invitrogen	Cat. 13778‐150
Hygromycin B	Invitrogen	Cat. 10687010
0.25 % Trypsin/EDTA	ThermoFisher	Cat. 25200‐056
DMEM GlutaMax 4.5 g/l D‐Glucose, Pyruvate	Thermo Fisher	Cat. 31966047
OPTIMEM medium	Invitrogen	Cat. 51985026
GFP‐Trap Agarose	YChromotek	Cat. GTA‐20
PhosSTOP Easypack	Roche	Cat. 04906837001
Thymidine	Sigma	Cat. T1895
IGEPAL CA‐630	Sigma	Cat. I8896
Protein Assay Dye Reagent Concentrate	Bio‐Rad	Cat. 5000006
NuPAGE LDS Sample buffer (4×)	Novex	Cat. NP0008
MOPS SDS Running Buffer (20×)	Invitrogen	Cat. NP0001‐02
cOmplete™ EDTA‐free Protease Inhibitor Cocktail	Roche	Cat. 4693132001
T4 polynucleotide kinase	Thermo Scientific	Cat. EK0031
T7 DNA polymerase	Thermo Scientific	Cat. EP0081
T4 DNA ligase	Thermo Scientific	Cat. EL0014
50 bp marker	Thermo Scientific	Cat. 10416014
Mag‐bind Total Pure NGS	Omega Bio‐tek	Cat. M1378‐01
QIAquick Gel extraction Kit	Qiagen	Cat. 28706X4
Quant‐iT PicoGreen dsDNA Assay Kit	Molecular probes by Life technologies	Cat. P7589
TMB substrate	Seracare KPL	Cat. 5120‐0047
QIAquick Nucleotide Removal Kit	Qiagen	Cat. 28306
GSH Sepharose 4 Fast Flow Media	Cytiva	Cat. 17513201
Ni Sepharose excel	Cytiva	Cat. 17371201
FastDigest SmaI	ThermoFisher	Cat. FD0663
Affinity chromatography columns, HiTrap™ Benzamidine FF	Cytiva	Cat. 17514302
Thrombin	Cytiva	Cat. 27‐0846‐01
Thermo Scientific™ Lysozyme	ThermoFisher	Cat. 89833
GelRed	Biotium	Cat. 41003‐T
Thermo Scientific™ Phusion High‐Fidelity PCR Master Mix with HF Buffer	ThermoFisher	Cat. F631L
QIAquick PCR Purification Kit	Qiagen	Cat. 28104
jetOPTIMUS® DNA transfection reagant and buffer	Polyplus	Cat. 101000006
96‐well Flat‐bottom Immunosorp MaxiSorp plates	Nunc, Roskilde, Denmark	Cat. 439454
384‐well Flat‐bottom Immunosorp MaxiSorp plates	Nunc, Roskilde, Denmark	Cat. 464718
96‐well half area black Flat‐bottom Nonbinding surface plates	Corning, USA	Cat. 3993
100 × 20 cm Greiner Cellstar Cell Culture Dish	Greiner Bio‐One	Cat. 664160
145 × 20 cm Greiner Cellstar Cell Culture Dish	Greiner Bio‐One	Cat. 639160
μ‐slide 8 well	Ibidi	Cat. 80826
**Software**		
GraphPad Prism version 9.3.1 for MacOS	GraphPad Software, San Diego, CA, USA, www.graphpad.com	
ImageJ	https://imagej.nih.gov/ij/	
PyMOL Version 2.1.1	New York, NY, USA Schrodinger LLC	
DeltaVision Softworx Software	DeltaVision	
Python version 3	Van Rossum and Drake (2009)	
Pandas	McKinney (2010)	
Matplotlib	Hunter (2007)	
Seaborn	Waskom *et al* (2021)	
Inkscape	Inkscape Project (2020). Inkscape. Retrieved from https://inkscape.org	
**Other**		
iD5	Molecular Devices	
iTC200	Malvern	
PCR machine	Biometra TGradient	
Illumina MiSeq v3 run, 1 × 150 bp read setup, 20% PhiX	NGS‐NGI SciLifeLab facility	
Nanodrop ND‐1000	Thermo Fisher	
DeltaVision Microscope	DeltaVision	

### Methods and Protocols

#### Peptides

Peptides were obtained either from Glbiochem (China) or Genecust (France) and dissolved in dimethyl sulphoxide (DMSO; for FITC‐labelled peptides) or 50 mM sodium phosphate buffer pH 7.4 (unlabelled peptides; Dataset EV8). For better solubility, some peptides were supplemented with 1–5 μl 1 M NaOH while monitoring that the pH does not exceed 8. The TPD52L2 peptides were as an exception supplemented with DMSO to improve solubility. The solubilised peptides were spun down for aggregates for 10 min at 4°C at 10,000 × *g*. Additionally, peptides were modified if needed to contain a C‐ or N‐terminal tyrosine to allow for concentration determination at 280 nm.

#### Protein expression and purification

*Escherichia coli BL21*‐Gold(DE3) bacteria were used as expression system. Bacteria were grown in 2YT medium (5 g/l NaCl, 16 g/l tryptone and 10 g/l yeast extract) to OD_600_ = 0.7, and protein expression was induced with 1 mM isopropyl β‐D‐1 thiogalactopyranoside (IPTG) for 18 h. The bacteria were pelleted for 15 min at 4,000 × *g* at 4°C and stored at −20°C. For purification, pellets were resuspended in lysis buffer (phosphate‐buffered saline (PBS) pH 7.4, 5 mg/ml MgCl_2_, 0.05% Triton‐X, lysozyme, 10 μg/ml DNAse and cOmplete™ EDTA‐free Protease Inhibitor Cocktail (Roche)), incubated for 1 h at 4°C and sonicated before spin down at 16,000 × *g* at 4°C. For phage selections, proteins were expressed as GST‐ or MBP‐tagged fusion proteins (Dataset EV3) and purified from the supernatant with glutathione (GSH) beads (Cytiva) or Ni^2+^ Excel beads (Cytiva), respectively.

For FP, ITC and crystallisation, the tag was removed with the appropriate protease either on beads (0.1 mg/ml thrombin (Cytiva) in PBS, 1 mM dithiothreitol (DTT)) or by dialysis in cleavage buffer (Human Rhinovirus (HRV) 3C protease: 50 mM Tris–HCl pH 7.4, 150 mM NaCl, 1 mM β‐mercaptoethanol), both procedures were overnight. In case of HRV protease cleavage, the tag was separated from the target protein domain by reverse ion metal affinity chromatography using Ni^2+^ beads for purification, whereas thrombin was removed using a benzamidine column (Cytiva). Affinity measurements for SNX27 PDZ were performed on the MBP‐tagged construct, as well as PIN1 WW domain on the GST‐tagged construct. For FP and ITC, proteins were dialysed in 50 mM sodium phosphate buffer pH 7.4, 1 mM DTT. For crystallisation of the NTD of clathrin, the protein was further purified by a S200 Sepharose column (Cytiva) using 20 mM Tris–HCl pH 7.4, 150 mM NaCl and 1 mM DTT.

#### Design of the PM_HD2 library

The PM_HD2 library was designed on the basis of our previously published HD2 library (Benz *et al*, 2022) by systematically mutating serine and threonine residues identified in a previously published MS‐defined phospho‐proteome (Ochoa *et al*, 2020) to glutamate. First, a list of phosphosites was built (Dataset EV2A). To keep the scale of the library to ~92,000 peptides, only serine and threonine phosphosites were considered and they were filtered by localisation probability (≥ 0.75) and functional score (≥ 0.4524). For proteins where none of its phosphosite surpassed the cut‐offs, the single phosphosite with the highest functional score was also kept. A list of proteins of interest was considered to rescue some phosphosites back, and these proteins of interest included KPNA4, a list of curated switches and ELM instances containing serine or threonine phosphosites (Dataset EV2B) and a list of curated NLS/NES (Dataset EV2C). Finally, this list of phosphosites was mapped onto the HD2 library design (Dataset EV2D) allowing only a single mutation at a time if multiple phosphosites occurred in the same peptide, and a wild‐type and a glutamate version of each peptide were reverse translated into oligonucleotides using optimised codons for *Escherichia coli* expression avoiding the formation of SmaI restriction sites (Dataset EV1). In the resulting design, most phosphosites are being covered by three or more overlapping peptides (Appendix Fig S1A) and there is an even distribution of the positions of the phosphosites along the 16 amino acids peptides (Appendix Fig S1B). Predicted kinases for all phosphosites in the PM_HD2 library were obtained from Ochoa *et al*, 2020 (Appendix Fig S1C), and phosphatases were obtained from DEPOD (Damle & Köhn, 2019).

#### Generation of the PM_HD2 library

The PM_HD2 library was, as previously described, generated on the p8 protein of the M13 bacteriophage by first amplifying the custom oligonucleotide array, annealing the amplified array to the single‐stranded phagemid which was followed by amplification of the phagemid (Kunkel reaction). After that, the library is transformed by electroporation into SS320 bacteria (Chen & Sidhu, 2014; Benz *et al*, 2022).

1. The custom oligonucleotide array (1 μl) was amplified with primers for internal p8 coat protein display and Phusion High‐Fidelity PCR Master Mix (Thermo Scientific).
2. Amplification was verified by running the PCR product on a 2% agarose gel and the PCR products pooled and cleaned up with QIAquick Nucleotide Removal Kit (Qiagen). The DNA content was quantified with Quant‐iT PicoGreen dsDNA Assay Kit.
3. The dsDNA product was denatured for 10 min at 98°C. Next, the amplified custom library (0.3 μg) was phosphorylated with the T4 polynucleotide kinase (10 units) for 1 h at 37°C. To the phosphorylated oligonucleotides, 10 μg dU‐ssDNA of the p8 phagemid was added (ratio 3:1) and the sample incubated for annealing 3 min at 90°C, 3 min at 50°C and last 5 min at 20°C.
4. For amplification of the phagemid, 30 units of T7 DNA polymerase and 30 Weiss units of the T4 DNA ligase were used and the sample incubated for 20 h at RT.
5. Wild‐type phagemid was removed by SmaI digestion (5 μl) for 30 min at 37°C. The CCC‐dsDNA was then purified QIAquick PCR Purification Kit and success of the Kunkel reaction verified by running the sample on a 2% agarose gel.
6. The library was transformed into electrocompetent SS320 bacteria (150 μl), which were preinfected with M13KO5 helper phages, and using a Gene Pulser (Bio‐Rad) electroporation system. The sample was immediately resuspended in 1 ml prewarmed SOC medium, transferred to additional 9 ml medium and incubated for 30 min at 37°C at 220 rpm. The 10 ml was transferred after that to a 500 ml culture containing 30 μg/ml kanamycin and 100 μg/ml carbenicillin and the phage library grown o.n. at 37°C at 220 rpm.
7. The bacteria were pelleted by centrifugation at 4,500 × *g* for 15 min at 4°C, the phages precipitated by addition of one‐fourth of the volume of 20% PEG‐800 and 0.4 M NaCl to the supernatant, incubation for 10 min on ice and subsequent spin at 16,000 × *g* for 10 min at 4°C. The pellet was resuspended in 20 ml 0.05% Tween‐20 in PBS, ultrapure glycerol added (10%) and the library stored at −80°C.

#### Phage display selections

Selections using the PM_HD2 phage library were performed as described previously with minor modification to the original protocol. The corresponding tag protein (either GST or MBP) was first used during negative selections. Selections were run for 4 days.

1. Bait and tag proteins (10–15 μg in 100 μl PBS) were immobilised in 96‐well MaxiSorb plate (NUNC) o.n. at 4°C.
2. *Escherichia coli* OmniMax bacteria were grown in 2YT medium supplemented with tetracycline (100 μg/ml) at 37°C and 200 rpm.
3. Plates with immobilised bait/tag proteins were blocked with 0.5% BSA in PBS for 1 h at 4°C.
4. Phage library (10 μl/well) was prepared by diluting 10× with PBS and adding one‐fourth of the volume of PEG/NaCl. For phage precipitation, prepared library was incubated for 10 min on ice followed by centrifugation for 10 min at 10,000 × *g* at 4°C. Phages were resuspended in 0.5% BSA, 0.05% Tween in PBS.
5. Plates with tag proteins were washed 4× with 0.05% Tween in PBS, and *naïve* phage library was added to each well (100 μl/well) and the plates incubated for 1 h at 4°C.
6. Plates with bait protein were washed 4× with 0.05% Tween in PBS, and nonbound phages were transferred from the tag protein plate and the plates incubated for 1 h at 4°C.
7. Plates were again washed 5× with 0.05% Tween in PBS, and bound phages were eluted with log‐phase OmniMax bacteria (100 μl/well) by incubating for 30 min at 37°C at 200 rpm.
8. M13K07 helper phage (10^12^ cfu/ml) was added to each well and the plates incubated for 45 min at 37°C at 200 rpm.
9. Amplification took place o.n. by adding the eluted phages to 1.1 ml 2YT media supplemented with kanamycin (25 μg/ml), carbenicillin (50 μg/ml) and 0.3 μM IPTG in a 96‐deep‐well plate and incubating o.n. at 37°C at 200 rpm.
10. Bacteria were pelleted by centrifugation for 15 min at 1,700 × *g* at 4°C. The phage supernatant was pH‐adjusted by the addition of 10× PBS and remaining bacteria heat‐inactivated by incubating for 10 min at 60°C. Selections were repeated as described from (5).

#### Phage Pool ELISA

1. Phage pool ELISA on selection Days 1–4 was performed as previously described (Chen & Sidhu, 2014; Benz *et al*, 2022). Briefly, bait domains and control proteins (5–7.5 μg of protein per well in 50 μl PBS) were immobilised in a 384‐well MaxiSorb plate (NUNC) overnight at 4°C while shaking.
2. Plates with immobilised protein were blocked with 0.5% BSA in PBS and incubated for 1 h at 4°C.
3. The blocking solution was removed, and the phage pools from the various selection days were added to the respective bait domains and their control protein, and the plates incubated for 1–2 h at 4°C while shaking.
4. The plates were washed 5× with 0.05% Tween in PBS, and 100 μl of anti‐M13 bacteriophage mouse antibody (Sino Biological Inc, 1:5,000) was added to each well and the plates incubated for 1 h at 4°C while shaking.
5. The antibody solution was washed off by washing the plates 4× with 0.05% Tween in PBS and 1× with PBS. To each well, 40 μl of TMB (3,3′,5,5′‐Tetramethylbenzidine) substrate was added and the enzymatic reaction subsequently stopped with 40 μl of 0.6 M H_2_SO_4_. The absorbance of bait protein domain and control protein at 450 nm was subsequently compared. The enrichment of phages was considered specific for the bait protein domain when the ratio at 450 nm was above 2.

#### Next‐generation sequencing (NGS)

Based on the results from phage pool ELISAs, samples from selection Day 3 and 4 were selected for NGS, as described previously (Benz *et al*, 2022).

1. Peptide coding regions were amplified and dual‐barcoded by PCR. 5 μl of the enriched phage pools (50 μl reactions) and a high‐fidelity phusion polymerase (Roche) were used for the PCR reaction. Amplification was confirmed by running the PCR product on 2% agarose gel with a 50 bp ladder.
2. PCR products (25 μl) were purified with 25 μl of Mag‐bind Total Pure NGS beads and normalised in the same step by taking 10 μl of each reaction after resuspension (Qiagen Elution buffer: 10 mM Tris‐Cl and 0.1 mM EDTA (pH 8.5)) from the beads.
3. The samples were cleaned up with a PCR purification kit (Qiagen) and subsequently run on a 2% agarose gel, and bands of the correct size (ca. 200 bp) were purified with the QIAquick Gel extraction Kit (Qiagen). From the columns, samples were eluted with 30 μl TE (10 mM Tris–HCl, 1 mM EDTA. pH 7.5) buffer and quantified with Quant‐iT PicoGreen dsDNA Assay Kit.
4. Samples were sequenced on MiSeq (MSC 2.5.0.5/RTA 1.18.54) with a 151 nt (Read1) setup using “Version3” chemistry. The Bcl to FastQ conversion was performed using bcl2fastq_v2.20.0.422 from the CASAVA software suite.

#### Sequencing analysis

The processing of sequencing data was performed as described in Ali *et al* (2020). In brief: Pooled results from ~500 selection experiments were demultiplexed by identifying unique combinations of 5′ and 3′ barcodes, sequences with an average quality score of 20 or more were conserved and their adaptors were trimmed, and DNA sequences translated using in‐house custom Python scripts. A table for each experiment was built containing peptide sequences associated with their sequencing counts.

#### Library coverage and quality

The completeness of the PM_HD2 library was evaluated by sequencing multiple nonchallenged *naïve* library aliquots. The observed coverage of the library was evaluated, and the maximum estimated coverage was determined (Appendix Fig S1D) as described in Benz *et al* (2022). Finally, the counts distribution per peptide was evaluated (Appendix Fig S1) and the percentage of wild‐type–mutant peptide pairs and their ratios distributions were assessed (Fig 1D). The sequence information (in terms of amino acid distribution) was largely unaffected between the HD2 library design, and the subset included in the PM_HD2 library (Appendix Fig S1G).

#### Selection results analysis

After building tables *per* experiment as described in the “Sequencing analysis” section, peptide sequences were analysed as described in Benz *et al* (2022) with some modifications regarding the evaluation of wild‐type vs. mutant versions of each peptide pair. In brief: Peptides with a read count of 1 and those peptides that did not match the library design were removed and the remaining peptide counts were normalised. Different selection days for the same replica experiment were merged, and their normalised counts average was calculated for each peptide. Different replicates for the same bait were joined together and PepTools was used to annotate all peptides and each peptide's confidence score was calculated as described previously (Benz *et al*, 2022). Finally, a mutation‐centred analysis was performed by evaluating the selection read counts for all wild‐type and mutant overlapping peptides for each phosphosite. This analysis included calculating a Mann–Whitney confidence score by comparing the counts from wild‐type vs mutated peptides and the calculation of the phosphomimetic enrichment score (*PES*) for the phosphosite following the equation:

(1) $$\mathrm{PES}=\sum_{i=0}^{n}\frac{{{\mathrm{nc}}^{i}}_{\mathrm{pm}}}{{{\mathrm{nc}}^{i}}_{\mathrm{wt}}+{{\mathrm{nc}}^{i}}_{\mathrm{pm}}}\;-\;\sum_{i=0}^{n}\frac{{{\mathrm{nc}}^{i}}_{\mathrm{wt}}}{{{\mathrm{nc}}^{i}}_{\mathrm{wt}}+{{\mathrm{nc}}^{i}}_{\mathrm{pm}}}$$

*PES* is calculated on the level of wild type (wt) and phosphomimetic mutation pair in selection against a given bait. The score includes data for overlapping peptide containing the same wild‐type and phosphomimetic pair. The *PES* is calculated using the normalised sequencing counts for the wild‐type $({{\mathrm{nc}}^{i}}_{\mathrm{wt}}$) and phosphomimetic $({{\mathrm{nc}}^{i}}_{\mathrm{pm}}$) data for all replicates for all peptides. Each wt and phosphomimetic mutation pair will have *n* datapoints, and the bounds of the score are ±*n*. A positive score indicates a phosphomimetic enhances binding, and a negative score indicates a phosphomimetic inhibits binding.

#### FP experiments

FP experiments were performed as described previously (Kliche *et al*, 2021). Shortly, in order to monitor the binding event, FITC‐labelled peptides were used (excitation 495 nm, emission: 535 nm) and the FP signal was recorded in mP on a SpectraMax iD5 Multi‐Mode Microplate Reader (Molecular Devices) by measuring the emitted light in two angels. The measurements were performed at room temperature and prepared in nonbinding black half area 96‐well microplates (Corning®; 50 μl/well). For measuring direct binding, that is saturation curves, a 1:1 dilution series of protein domain (25 μl/well) was generated in 50 mM sodium phosphate buffer pH 7.4 and the measurement performed with 5 nM FITC‐labelled peptide (25 μl/well). The buffer‐corrected values were subsequently plotted with PRISM and fitted to the quadratic equation (Gianni *et al*, 2005).

In order to record displacement curves, a complex of protein domain ([domain] = 1–2 fold over K_D_ of FITC‐labelled peptide) and FITC‐labelled peptide (10 nM in complex, 5 nM in the measurement) was used. The 1:1 dilution series in this experiment was performed with the unlabelled peptide in 50 mM sodium phosphate buffer pH 7.4, so that each well‐contained unlabelled peptide (25 μl) at a certain concentration and pre‐formed complex (25 μl). The K_D_ values of this indirectly monitored binding event were calculated as described previously (Nikolovska‐Coleska *et al*, 2004). All measurements were at least in technical triplicates as well as biological duplicates when indicated. An amplitude shift between repeat measurements in the displacement experiments was occasionally observed due to variations in the protein preparations. If that was the case, the “active” protein concentration was back‐calculated according to the quadratic equation from the saturation curves performed in parallel. By comparing the active protein concentrations in the complex between different protein preparations, the amplitude was normalised. In addition, raw FP values were checked for FITC‐contamination of the unlabelled peptides, which resulted in the omission of the data and repeat measurements with newly ordered peptides.

#### ITC experiments

Both peptide and protein domains were dialysed in the same buffer to avoid buffer mismatch causing artefacts (50 mM sodium phosphate buffer pH 7.4, 1 mM DTT). Experiments were performed on an iTC200 instrument (Malvern) by titrating peptides against protein domains in approximately 10‐fold excess (16 injections). The provided programme on the instrument was used for curve fitting, and manual baseline correction was applied if suitable. Technical triplicates were obtained for all measurements, except for the GGA1 VHS domain binding to the RNF11 peptide for which a technical duplicate has been obtained.

#### Crystallisation

For crystallisation, clathrin NTD (19 mg/ml) and HURP pS839 C‐terminal peptide (6.4 mM in 50 mM sodium phosphate buffer pH 7.4) were mixed in 1:1 molar ratio. Initially, the crystallisation was attempted by using reported crystallisation condition (50 mM Tris pH‐7.5 and 30% PEG 6000; Rondelet *et al*, 2020). The hanging drop method was applied, and correspondingly, 2 μl of complex was mixed with 2 μl of reservoir solution of composition 24% PEG‐6000, 100 mM Tris–HCl pH 7.5 and pH 8.0. The crystallisation was performed at 293 K and crystals grew within 3 days. CLTC‐NTD complex crystals were cryoprotected in mother liquor supplemented with 20% glycerol and flash‐frozen in liquid nitrogen.

#### X‐ray data collection, structure determination and refinement

For the two peptide complexes of CLTC‐NTD, crystallographic data were collected at 100 K at the beamline ID‐23‐1 at European synchrotron Radiation Facility (Grenoble, France) and autoindexed and processed using Xia2 (Winter & McAuley, 2011). The structures were solved by molecular replacement using Phaser (McCoy *et al*, 2007) and PDB entry 1C9I as search model (ter Haar *et al*, 2000). Structure was refined with phenix.refine and Refmac5 of the Phenix (Adams *et al*, 2010) and CCP4 program suites (Winn *et al*, 2011), respectively. Manual model building was done in COOT (Emsley & Cowtan, 2004). The final structures showed good geometry as analysed by Molprobity (Chen *et al*, 2010). The data collection and refinement statistics are given in Dataset EV11.

#### Generation of stable cell lines

YFP‐HURP WT and Δmut (Δ835–846) constructs were generated via the insertion of HURP with BamHI/NotI into YFP‐pCDNA/FRT/TO; in addition, S839A HURP was generated by site‐directed mutagenesis. RNAi‐resistant constructs were further generated using primers as listed in the reagent and tools table. Flp‐In T‐REx HeLa cells were co‐transfected with pOG44 (4.5 μg) and pcDNA5/FRT/TO containing the different YFP‐tagged versions of HURP (0.5 μg; wild‐type, Δmut and S839A HURP) in 0.5 ml of jetOPTIMUS® buffer and 10 μl of jetOPTIMUS® DNA Transfection Reagent (Polyplus). After 24 h, the medium was changed to selection medium (DMEM 10% FBS, 1% penicillin/streptomycin, 5 μg/ml blasticidin, 0.1 mg/ml hygromycin) to select for cells stably expressing the protein of interest.

#### Co‐immunoprecipitation

For Co‐IP experiments, HeLa cells inducible expressing either YFP‐tagged HURP_WT_, HURP_ΔMUT_, HURP_S839A_ or YFP only were cultured in selection medium at 37°C, 5% CO_2_ in a humid environment. Cells were synchronised by 24 h incubation with 2.5 mM thymidine and either collected (early S phase) or released with 200 ng/ml nocodazole (mitotic). Mitotic cells were collected by shake‐off after 20 h. Expression was induced for both asynchronous and mitotic cells for 24 h at 5 ng/ml doxycycline. After collection, cells were resuspended in lysis buffer (100 mM NaCl, 50 mM Tris–HCl pH 7.4, 0.05% NP‐40, 1 mM DTT and completed with phosphatase (PhosSTOP (Roche)) and protease (cOmplete EDTA‐free (Roche)) inhibitors) and subsequently lysed by sonication. Lysate was cleared by centrifugation at 20,000 × *g* at 4°C for 45 min. GFP‐trap beads (Chromotek) were used for pulldown and incubated with supernatant for 1 h at 4°C on a rotating wheel. After that, beads were washed (150 mM NaCl, 50 mM Tris–HCl pH 7.4, 0.05% NP‐40, 1 mM DTT and completed with phosphatase and protease inhibitors as above) and eluted with twofold NuPAGE LDS Sample buffer (Novex). Samples were separated by SDS–PAGE and blotted in transfer buffer (25 mM Tris, 192 mM glycine, 20% ethanol) at 200 mA at 4°C for 3 h. Primary antibodies (anti‐CHC; anti‐HURP; anti‐GFP) were diluted 1:1,000 in 2.5% milk PBST and incubated o.n. at 4°C. Blots were washed with PBST (PBS and 0.1% Tween‐20), incubated with secondary antibodies (IRDye 800CW anti‐mouse; IRDye 680RD anti‐rabbit) for 1 h at room temperature and washed again. For visualisation, Odyssey CLx (LI‐COR) was used. For the S839A HURP experiments, the salt was reduced in both lysis and wash buffer to 50 mM NaCl and the anti‐CHC antibody in a dilution of 1:400.

#### RNA interference (RNAi) rescue experiments

For the knockdown experiments, HeLa cell lines inducible expressing HURP_WT_, HURP_ΔMUT_, HURP_S839A_ or YFP only were maintained under the same conditions as for the co‐immunoprecipitation and transfected with either HURP or control (Luciferase) siRNA (10 nM final concentration) using Lipofectamine™ RNAiMAX Transfection Reagent (Invitrogen) in OPTIMEM medium (Invitrogen), which was supplemented after 6 h with 10% FBS. Medium was changed to selection medium after 24 h, and again after additional 24 h, cells were seeded at 60% in ibidi dishes in selection medium and synchronised for 24 h with 2.5 mM thymidine. The cells were washed to release them from the thymidine block, and medium was changed to L15 medium with 10% FBS and 1 ng/μl doxycycline. The ibidi dishes were mounted for time‐lapse microscopy 2–4 h after induction on a Delta Vision microscope (Leica). Mitotic progression was monitored by taking images every 5 min for 18 h at different areas of the wells and three z‐stacks (5 μm apart). Image analysis was done with Deltavision Softworx Software and Fiji.

## Author contributions

**Johanna Kliche:** Conceptualization; data curation; investigation; visualization; writing – original draft; writing – review and editing. **Dimitriya Hristoforova Garvanska:** Investigation; visualization; writing – review and editing. **Leandro Simonetti:** Data curation; formal analysis; investigation; visualization; writing – review and editing. **Dilip Badgujar:** Investigation; writing – review and editing. **Doreen Dobritzsch:** Formal analysis; supervision; writing – review and editing. **Jakob Nilsson:** Supervision; investigation; writing – review and editing. **Norman E Davey:** Conceptualization; data curation; investigation; visualization; writing – original draft; writing – review and editing. **Ylva Ivarsson:** Conceptualization; funding acquisition; investigation; writing – original draft; project administration; writing – review and editing.

## Disclosure and competing interests statement

The authors declare that they have no conflict of interest.

## Supporting information



Appendix

Dataset EV1

Dataset EV2

Dataset EV3

Dataset EV4

Dataset EV5

Dataset EV6

Dataset EV7

Dataset EV8

Dataset EV9

Dataset EV10

Dataset EV11

Source Data for Appendix

Source Data for Figure 6

## Data Availability

The datasets produced in this study are available in the following databases:
Crystal structure: PDB (https://www.rcsb.org/structure/7ZX4). Protein–protein interaction data: submitted to IMEx consortium through IntAct under the identifier IM‐29716 (http://www.imexconsortium.org). ProP‐PD data: ProP‐PD database (http://slim.icr.ac.uk/proppd/).
