# Supplementary material for: Large‐scale phosphomimetic screening identifies phospho‐modulated motif‐based protein interactions
Source: Mol Syst Biol. 2023 May 23;19(7):e11164. doi: 10.15252/msb.202211164 (PMC10333884; doi:10.15252/msb.202211164)
Supplement: Supplementary file 1 — Appendix [file MSB-19-e11164-s004.pdf]

Large-scale phosphomimetic screening identifies phospho-modulated motif-based protein interactions

Johanna Kliche[1], Dimitriya Hristoforova Garvanska[2], Leandro Simonetti[1], Dilip Badgujar[1], Doreen Dobritzsch[1], Jakob Nilsson[2], Norman E. Davey[3,*] & Ylva Ivarsson[1;*]

Table of contents: Appendix figures

Appendix Figure S1: PM_HD2 library design and validation…………………………….Page 2

Appendix Figure S2: FP displacement experiment of obligate phospho-binders……..Page 4

Appendix Figure S3: Saturation experiments of protein domains and FITC-labelled peptides…………………………………………………………………………………....Page 4

Appendix Figure S4: FP displacement experiments from the different protein domains…………………………………………………………………………………….Page 5

Appendix Figure S5: ITC curves…………………………………………………………....Page 6

Appendix Figure S6: Comparison of clathrin binding to different LIxF-peptides…...…Page 7

Appendix figures

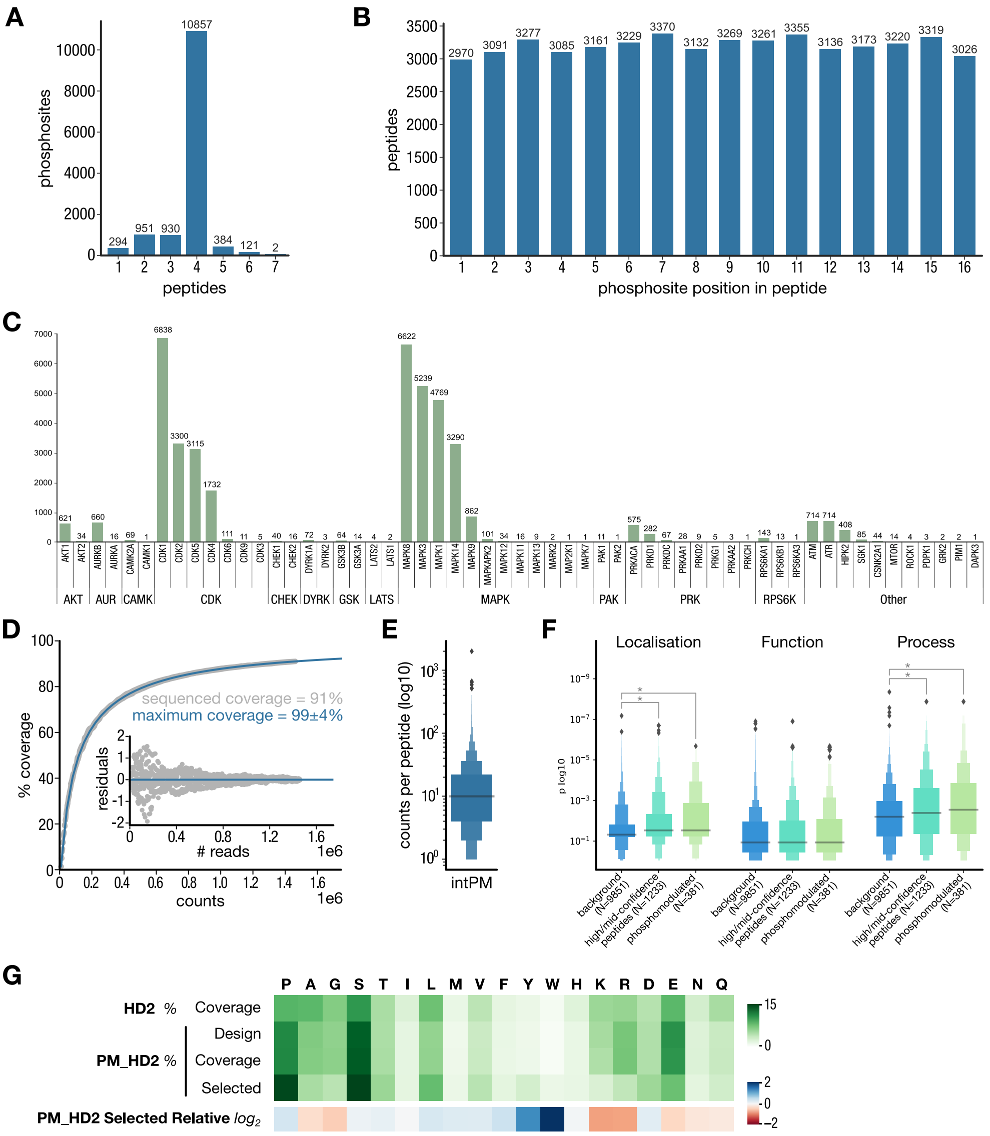

Appendix Figure S1: PM_HD2 library design and quality. A. Number of peptides tiling each phosphosite. **B**. Distribution of phosphosite positions among the 16 amino acid long peptides. **C.** Number of predicted kinases for all phosphosites in the library. **D.** Peptide coverage obtained after sequencing of the PM_HD2 library. The maximum coverage was calculated by fitting the coverage percent vs. number of reads to a double hyperbolic curve as described in Benz *et al.* (2022). **E.** Counts per peptide distribution in the *naïve* sequencing of the PM_HD2 library. **F:** Distribution of the p-values for the most significant shared GO terms. The background group consists of all bait-peptide pairs with confidence of 1 or lower, the high/mid-confidence group consists of all interacting bait-peptide pairs with confidence level of 2 or more, and the third (putative phospho-modulated) group corresponds to the sub-set of

high/mid-confidence protein-bait interactions that is suggested to be phospho-modulated according to this study ($p \leq 0.01$ and PES ≥ 2). Asterisk (*) denotes Mann-Whitney U test p-value less than 0.001. Note that the phage selection enriched for peptides from proteins sharing GO terms related to localization and function with the bait. **G:** Distribution of amino acids in the PM_HD2 library at the design, coverage and selection levels, together with the log2 fold change between selection and coverage are shown. The amino acid percentages for HD2 at coverage level are also shown (Benz *et al*, 2022)

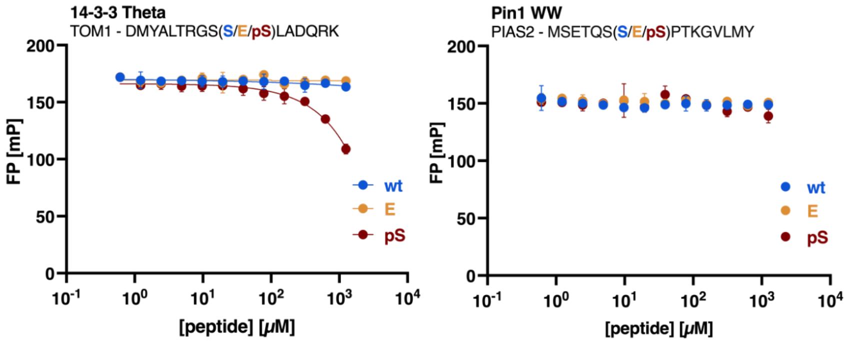

Appendix Figure S2: **FP displacement experiment of obligate phospho-binders**. Displacement curves from FP experiments of 14-3-3 Theta and PIN1 WW domain with wild-type, phosphomimetic and phosphorylated peptides from TOM1 and PIAS2, respectively. Measurements were in three technical triplicates.

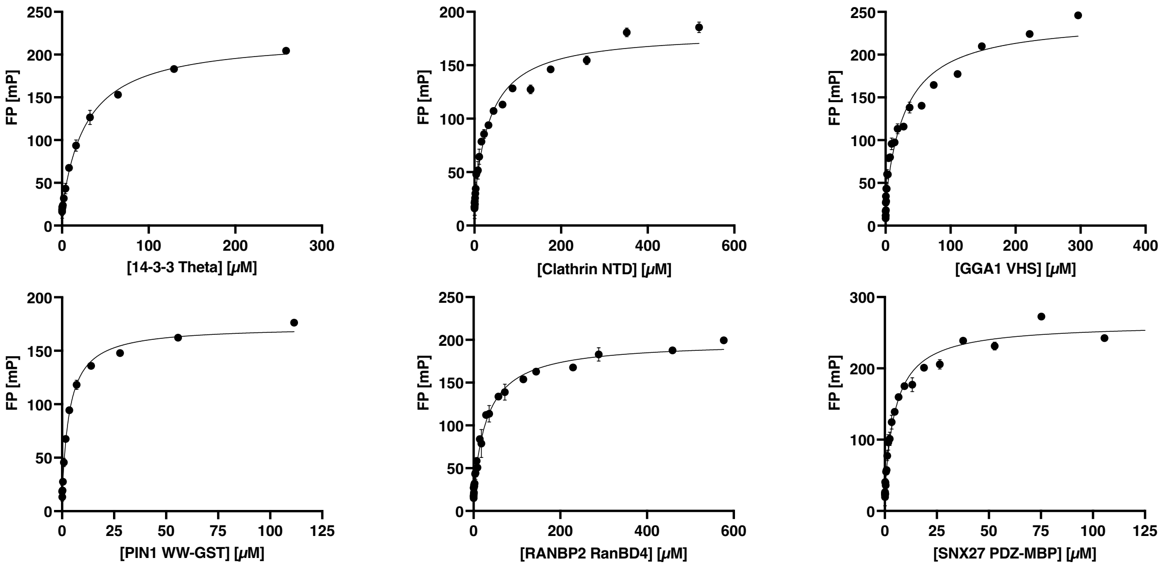

Appendix Figure S3: **Saturation experiments of protein domains and FITC-labelled peptides**. Detailed information on peptides is provided in **Dataset** EV8. Measurements were in at least technical triplicates.

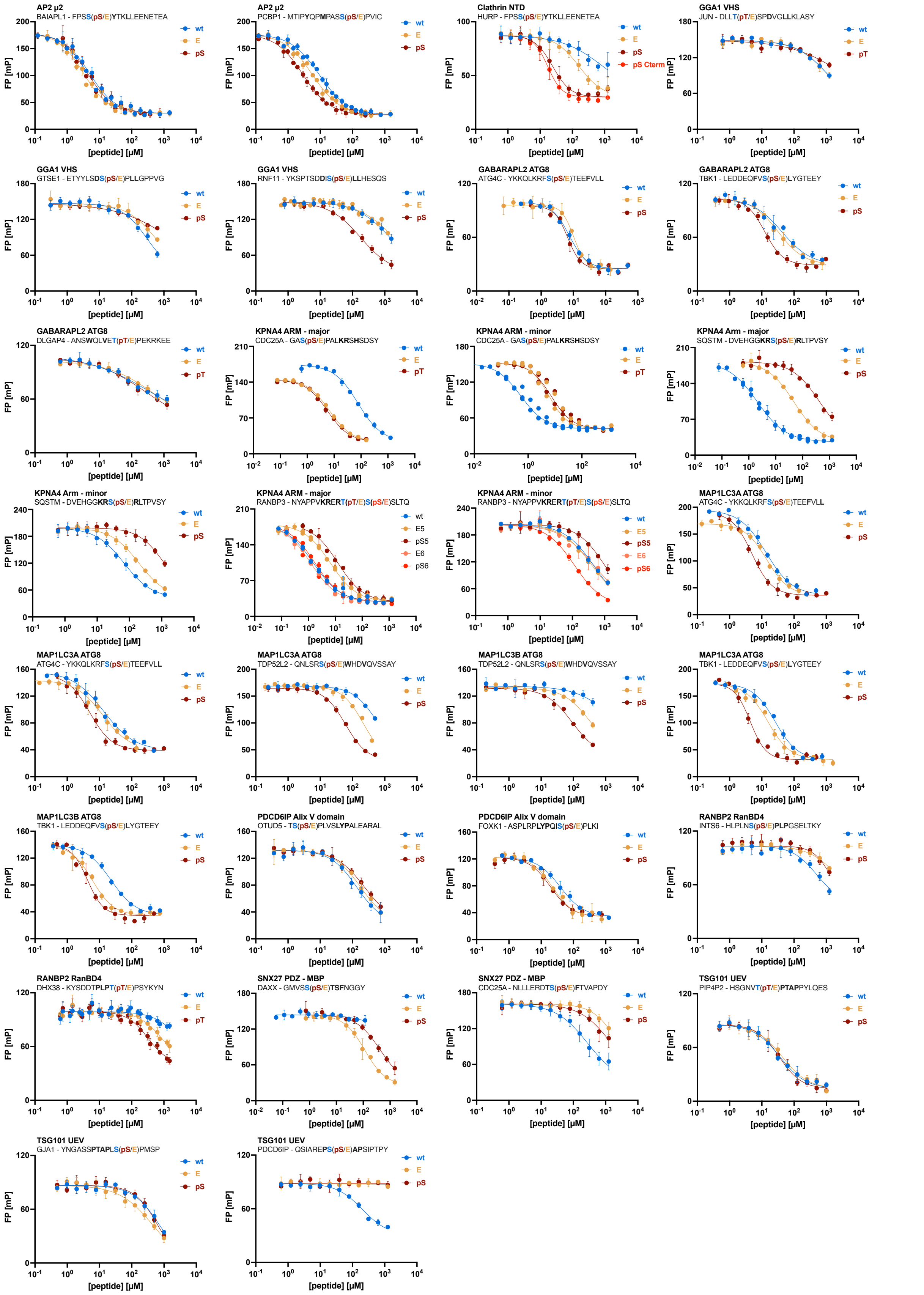

Appendix Figure S4: FP displacement experiments from the different protein domains. Displacement curves of the different protein domains and the peptide triplets (wild-type, phosphomimetic and phosphorylated). Measurements were in technical triplicates.

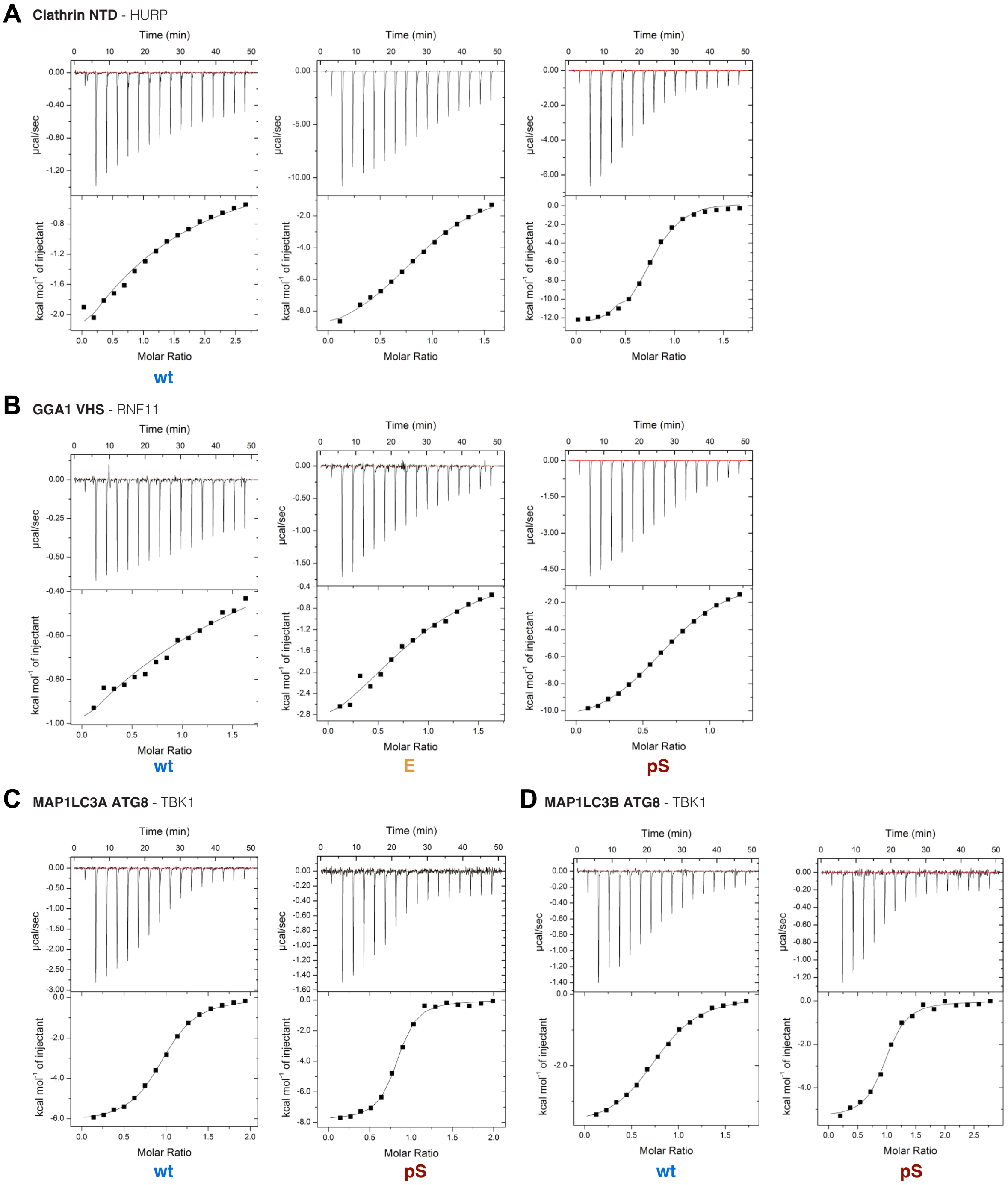

Appendix Figure S5: ITC curves. ITC curves of Clathrin NTD binding to the HURP peptide (wild-type, phosphomimetic and phosphorylated), GGA1 VHS binding to the RNF11 peptide (wild-type, phosphomimetic and phosphorylated) and the ATG8 proteins MAP1LC3A and -B binding to the TBK1 peptide (wild-type and phosphorylated). Measurements were in technical triplicates, except for the GGA1 VHS binding curves which were in duplicates.

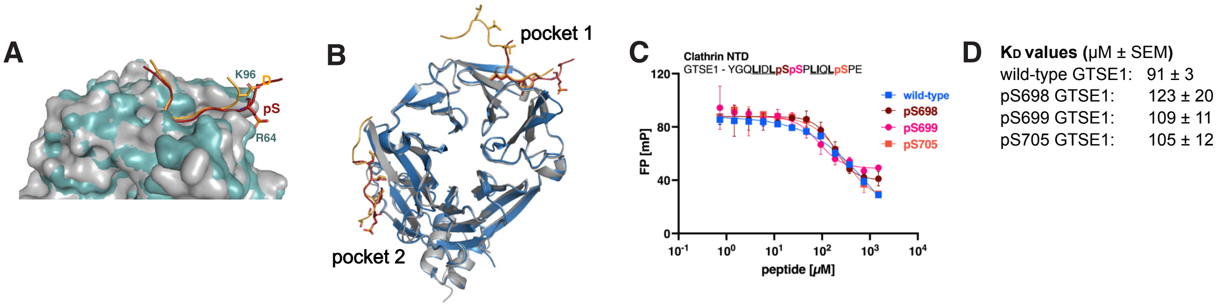

Appendix Figure S6: Comparison of clathrin binding to different LIxF-peptides. **A.** Superimposition of clathrin NTD structure bound to non-phosphorylated (PDB: 1C9I) and phosphorylated peptide (this study). Comparison of the orientation of the residue at the p+5 position on the peptide, which is either an aspartate (1C9I) or the phosphorylated serine (this study). B: Superimposition of clathrin NTD bound to peptides from HURP (this study) or GTSE1 (PDB: 6QNP) (Rondelet *et al*, 2020)). **C**: Displacement curves from FP experiments of clathrin NTD with GTSE1 peptides with varying phosphorylation. Measurements were in technical triplicates. **D**: K_D-values and SEM from the displacement experiments of clathrin NTD with GTSE1 peptides.
