# Supplementary material for: Large‐scale phosphomimetic screening identifies phospho‐modulated motif‐based protein interactions
Source: Mol Syst Biol. 2023 May 23;19(7):e11164. doi: 10.15252/msb.202211164 (PMC10333884; doi:10.15252/msb.202211164)
Supplement: Supplementary file 15 [file MSB-19-e11164-s009.pdf]

molecular systems biology

Large-scale phosphomimetic screening identifies phospho-modulated motif-based protein interactions

Johanna Kliche, Dimitriya Garvanska, Leandro Simonetti, Dilip Badgujar, Doreen Dobritzsch, Jakob Nilsson, Norman Davey, and Ylva Ivarsson
DOI: 10.15252/msb.202211164

Corresponding author(s): Ylva Ivarsson (ylva.ivarsson@kemi.uu.se) , Norman Davey (norman.davey@icr.ac.uk)

Review Timeline:

Submission Date:	8th Jun 22
Editorial Decision:	21st Jul 22
Appeal Received:	14th Feb 23
Editorial Decision:	22nd Mar 23
Revision Received:	24th Apr 23
Editorial Decision:	28th Apr 23
Revision Received:	2nd May 23
Accepted:	3rd May 23

Editor: Maria Polychronidou

Transaction Report:

(Note: With the exception of the correction of typographical or spelling errors that could be a source of ambiguity, letters and reports are not edited. Depending on transfer agreements, referee reports obtained elsewhere may or may not be included in this compilation. Referee reports are anonymous unless the Referee chooses to sign their reports.)

21st Jul 2022

RE: Manuscript MSB-2022-11164, Large-scale identification of phospho-modulated motif-based protein-protein interactions

Dear Ylva,

Thank you again for submitting your work to Molecular Systems Biology. I apologise for the delay in sending you a decision, which was due to the fact that I was out of office for the last two weeks. We have now heard back from the three referees who agreed to evaluate your study. As you will see below, the reviewers raise substantial concerns on your work, which unfortunately preclude its publication in Molecular Systems Biology.

The reviewers appreciate that the addressed topic is relevant. However, they feel that the novelty remains limited and they raise significant concerns related to the data analysis/interpretation and the validity of the conclusions. As such, the reviewers rated the novelty and conclusiveness of the study as "Low/Medium" and indicated that they do not support publication in Molecular Systems Biology.

Taken together and given the rather low level of support provided by the reviewers, I am afraid I see no other choice than to return the manuscript with the message that we cannot offer to publish it. I am sorry that the review of your work did not result in a more favorable outcome on this occasion, and I hope that you will not be discouraged from submitting future work to Molecular Systems Biology. In any case, thank you for the opportunity to examine this work.

Best wishes,

Maria

Maria Polychronidou, PhD
Senior Editor
Molecular Systems Biology

Reviewer #1:

This paper is a well-written and describes a systematic approach to screening for protein domain-motif ligand interactions that may be regulated by phosphorylation. The authors use phage display and a few existing tools and libraries to arrive at an experiment to measure the interaction between 73 domains and 13,539 peptides. The peptides are presented as wild type (containing a serine/threonine) and as mutants where the S/T are changed to glutamic acid. Ultimately, the work is testing the sensitivity to a charged amino acid substitution, which is limited and recapitulates like other studies have that these mimics are poor substitutes for phosphorylation-driven interactions (i.e. phosphorylation obligate interactions like with WW and 14-3-3 domains). However, the authors are clear about this limitation and they go on to test phosphorylated peptides in downstream analyses for a few key phospho-regulated interactions identified by the study. The study is interesting and rigorous (following through to check the phosphorylation status of key interactions).

Summary of experiments and results:
988,347 interactions were tested (73 domains x 13,539 peptide pairs)
1,070 were identified as interactions
252 were regulated by the mutation
32 were tested by an FP displacement assay (both phosphorylated peptides and phosphomimetic)

• It seems surprising that for a peptide library selected specifically to cover disordered segments and phosphorylation sites "likely to participate in PPIs" that only 0.1% of possible interactions (of the wild type library, not accounting for individual interactions tested for the mutant library) produced an experimental interaction. It seems important for the authors to give some context as to why this is. For example, is there evidence that the Ochoa et al. constraints did more or less than what might be expected by random chance? Is the role of pS and pT really not to interact/modulate protein-protein interactions? Or was the criteria for selecting an interaction overly stringent and there are more interactions than reported at a different stringency level?
o Related to this - what is the likely overlap between domains and phosphosites in a physiological context? The manuscript states that the domains are from diverse proteins, but is there any indication that there might be network overlap between the library and the domains? For example, do protein-protein interaction databases place these two partners within some relevant signaling context or proximity in the network?

• I would have liked a better understanding of the library as designed. Specifically, I wished to understand better the following details:
o Given the tiling approach, what is the distribution of positions the phosphosite occupies relative to the ends? Did the HD2 combination with Ochoa selection also steer the library to centralize the phosphosite, or are some or many sites near the end of a peptide?
o How did selection criteria change/alter the sequence distributions of the library? For example, given the phosphoproteome as a whole, the starting HD2 library that has phosphorylation sites, and then the final library that uses the Ochoa constraints - does sequence information remain similarly diverse or does it become increasingly constrained?
o A majority of the phosphosites have annotations for kinases, which is a true enrichment, compared to the general phosphoproteome - what is the diversity of kinases connected to these sites? I could see concern for skewing the library towards particular kinases that are well studied/understood and could contribute to a constraint on what the library covered that would be helpful for readers to understand.
• One of the challenges of high throughput binding assays such as this is the protein purification and functional assessment of the domains produced. For example, in the obligate pairs tested with phosphosites, no positive indication of binding was seen for phosphopeptides, suggesting the domain may not have been functional. It would be helpful if the authors could indicate a positive control interaction that was expected for every domain tested and whether it was observed. The lack of observation might suggest which of the domains should be dropped from consideration having demonstrated no function at any point in the experiments. Although the authors make no indication in using or trusting the negative data from this experiment, others in the field may do so and so it could be very helpful to indicate experiments of dubiousness based on domains that gave no positive result.
• In the final experiments that take a phosphomodulated motif described into cells this experiment tests the overall interaction (focusing on deleting the 12 amino acids that define the ligand) and not the role for phosphomodulation in regulating the interaction, which seems a major limitation. Given the focus of the method leading up to this it seems more relevant to make point mutations of the phosphosite to phosphomimetic and a non-phosphorylatable amino acid that conserves the motif to truly connect the role of phosphomodulation to the cell cycle and mitosis.

Minor comments:
• Where peptide bounds are given, it would be helpful to also specify the site of phosphorylation of study in the given target. (e.g. TOM1366-382 which is the phosphosite?)
• The authors refer to "promiscuous phosphorylation" in the introduction, which is not a scientific term and (in my opinion) used lazily by scientists to talk about molecular biology that we do not fully understand. I don't see the point of using this term, it detracts for the science and in some cultures is offensive. Instead, it would be better to clearly delineate what is meant here - is it truly that there are non-functional phosphorylation sites that are tolerated because they are netural?

Reviewer #2:

Review on "Large-scale identification of phospho-modulated motif-based protein-protein

Kliche et al report on a phage display approach with 70 protein domains screening a libarary of 90000 peptides that cover regions of IDs. These ID regions are known to be post translationally modified/ phosphorylated in patches /cluster (literature is missing). Therefore, the authors include X to E substitutions that resemble 13000 phospho-sites from 6200 proteins in the peptide library. The claim is that this way phospho-modulated motif-based PPIs can be studied on a large scale.
1070 interactions were identified involving 52 domains where 126 PPIs showed a decreased and 126 showed an increased signal with an E substitution. Notably, selections against phospho-serine/threonine-binding domains failed. Individual pairs are tested through displacement binding experiments with synthesized phospho-peptides in use for comparison. I would argue with mixed outcome, which includes low affinities and various diverse effects. For three pairs a motif is scanned with E and pS peptides, again with three different effects. Finally, biological validation of Clathrin binding to a C-terminal HURP peptide motif was found to be cell cycle condition dependent. These experiments are well conducted and assay the peptide motif through deletion of the C-terminal motif. Ser839 is the proposed target for CDKs in the mitotic phase of cells.

Points for consideration
*) Figure 3 is key to the study. Here a volcano plot shows the differences between tiling X to E peptides in binding as determined by sequencing. However, it is clear from the plot that the data analysis is somehow flawed. The P-value is a linear function of the read differences and thus not a measure of statistical significance. I guess this is because of data processing that does not meet high quality criteria. First, the cut -off for taking a sequence read into consideration is 1! Second, there is no background i.e. sequencing from non selective condition taken into the equation. So enrichment is not quantified. Third, all sequences that represent one experiment (i.e. domain) are pooled, so that there are essentially no replicas of the experiment. With one value (I guess absolute read count) it is actually very difficult to make any statistical evaluation of the screen.
*) "Overall, the analysis supports that the phosphomimetic mutation is a valid proxy for serine/threonine phosphorylation and tool for probing the effects of phospho-modulation on interactions"
The study does not convincingly demonstrate that what we are looking at is actually about phosphorylation. Linear Peptide motifs are binding signatures that per definition tell about the influence of amino acid exchanges, including the exchange to E.

Any change of amino acid may have an effect (reflected in the motif as such) and for example alter affinities (This is actually concluded in Figure 4A). The potential of how phosphorylation affects motif signature has been studied in a couple of works e.g. by J Reimand et al.. Here it would be important to demonstrate that phosphorylation (as reversible regulatory "mutation") plays a special role in this context, e.g. by frequency, effect size or that phospho-recognition is special.
However, the data do not support this: phospho-binding domains largely failed, and the individual cases studied give a set of diverse results (Figure 3B, Figure S4). E.g. GGA1VHS three peptides would represent such a result, where in one case phosphorylation can be important in the other case it is maybe about the mutation. So what if the phospho-peptide has a smaller effect than the mutation and vice versa? Several of the pairs that meet the cut off do not show differences in binding of both the mutation and the phospho-version.
What about fully mutagenizing the phospho-sites and see if E/D is actually different than any other mutation (that violates the motif info)?
*) Figure 6: The interaction is clearly depended on synchronizing the cell and nocodazole release. However it is unclear why the control experiments was performed with a C-term deletion and not e.g. with S839A or S839E mutant versions of the proteins. This would much better reflect the approach of the paper and could demonstrate that the non-phosphorylate version S839A is not binding.

Reviewer #3:

Kliche et al. performed a large-sale study to identify phospho-regulated protein-protein interactions using their previously established proteomic peptide-phage display (Prop-PD) method. Overall, they screened 73 modular domains and identified 252 putative phosphor-modulated interactions. They further performed affinity analyses on a subset of 21 interactions. Finally, they studied a novel phospho-dependent interaction between clathrin and the mitotic spindle protein HURP in detail using various biophysical, molecular assays and analyses.

Major:
1. My main criticism is that the current study seems to be a scale-up from the authors' previous small-scale study from 2018 in the same journal (Sundell et al. Mol Syst Biol) to identify phosho-regulated interactions for PDZ domains.
2. I recommend the authors to develop a user-friendly webserver to the present their data so that other researchers in the field can tactfully explore their results.
3. Recent reports suggest that Histidine phosphorylation may play critical roles in the cell (Hindupur et al. Nature, 555, pages678-682 (2018); Fuhs and Hunter Curr. Opin. Cell Biol. 45, 8-16 (2017). However, very recently, Leijten et al (Nature Methods, 2022 https://www.nature.com/articles/s41592-022-01524-0) reported "we do not find any evidence in our data supporting that protein histidine phosphorylation plays a role in mammalian signaling". Can the authors comment on their understanding of the role of Histidine phosphorylation in cellular functions? Can they also discuss if they made any efforts to study the role of Histidine phosphorylation in regulating protein-protein interactions?

Minor:

1. What was the criteria for selecting 32 interactions for the affinity analysis?

2. The authors need to discuss potential limitations of their approach in understanding phosphor-mediated protein-protein interactions.

** As a service to authors, EMBO Press offers the possibility to directly transfer declined manuscripts to another EMBO Press title or to the open access journal Life Science Alliance launched in partnership between EMBO Press, Rockefeller University Press and Cold Spring Harbor Laboratory Press. The full manuscript and if applicable, reviewers' reports, are automatically sent to the receiving journal to allow for fast handling and a prompt decision on your manuscript. For more details of this service, and to transfer your manuscript please click on Link Not Available. **

Reply to reviewers' comments

Reviewer #1

This paper is a well-written and describes a systematic approach to screening for protein domain-motif ligand interactions that may be regulated by phosphorylation. The authors use phage display and a few existing tools and libraries to arrive at an experiment to measure the interaction between 73 domains and 13,539 peptides. The peptides are presented as wild type (containing a serine/threonine) and as mutants where the S/T are changed to glutamic acid. Ultimately, the work is testing the sensitivity to a charged amino acid substitution, which is limited and recapitulates like other studies have that these mimics are poor substitutes for phosphorylation-driven interactions (i.e. phosphorylation obligate interactions like with WW and 14-3-3 domains). However, the authors are clear about this limitation and they go on to test phosphorylated peptides in downstream analyses for a few key phospho-regulated interactions identified by the study. The study is interesting and rigorous (following through to check the phosphorylation status of key interactions).

Summary of experiments and results:
988,347 interactions were tested (73 domains x 13,539 peptide pairs)
1,070 were identified as interactions
252 were regulated by the mutation
32 were tested by an FP displacement assay (both phosphorylated peptides and phosphomimetic)

Question 1:
It seems surprising that for a peptide library selected specifically to cover disordered segments and phosphorylation sites "likely to participate in PPIs" that only 0.1% of possible interactions (of the wild type library, not accounting for individual interactions tested for the mutant library) produced an experimental interaction. It seems important for the authors to give some context as to why this is. For example, is there evidence that the Ochoa et al. constraints did more or less than what might be expected by random chance?
Is the role of pS and pT really not to interact/modulate protein-protein interactions? Or was the criteria for selecting an interaction overly stringent and there are more interactions than reported at a different stringency level?

Reply:
It is a fair question that we can break down into two parts: i) is the number of SLiM-based interactions reasonable, and ii) are the numbers of identified phosphosites that modulate these interactions reasonable.

To answer the first question, we screened 71 (73 if including the negative controls) domains for SLiM-based interactions. We identified 895 interactions, which is a reasonable number given the limited library size and the limitations set by the domain collection. For comparison, we released about 2,000 interactions for 35 proteins found by screening our human disorderome library that contains 1 million peptides (Benz et al., 2022).
The criteria for assigning confidence to the interactions have been carefully benchmarked before (see Benz et al., 2022). Clearly, if we relax the criteria, we will retain more real interactions, but also report many more false positive interactions. For the sanity of the field, we find it wiser to report fewer but higher confidence interactions.

To answer the second question. We find the number of interactions affected the phosphomimetic mutations, and in extension are suggested to be phospho-modulated,

reasonable. Importantly, the number of interactions and phospho-modulated interactions reported in the study does not report on the total number of functional phosphosites in the library. To start with, the results do not report on interactions that are strictly dependent on ligand phosphorylation, but rather on interactions that are modulated by phosphorylation. Secondly, given that there are thousands of SLiM-binding domains in the human proteome and we only sample around 70 domains, we only explore a fraction of interactions that these peptides may engage in, and consequently only a fraction of the phospho-modulated interactions. Finally, phosphorylation of IDRs may of course have other functions than modulating affinities of SLiM-based interactions, which we have clarified in the text (see discussion).

Regarding the Ochoa et al study, we find that that the quality of that study speaks for itself in terms of validations of filtering for functional sites, as the authors showed that the scoring ranked known functional phosphosites higher than the overall background and they also functionally validated several of the prioritized phospho-sites.

Question 2:
Related to this - what is the likely overlap between domains and phosphosites in a physiological context? The manuscript states that the domains are from diverse proteins, but is there any indication that there might be network overlap between the library and the domains? For example, do protein-protein interaction databases place these two partners within some relevant signaling context or proximity in the network?

Reply:
The proteins represented by peptides in the library are from 6247 different proteins, which represent about 30% of the proteins in the proteome. When looking at the binding enriched peptides, we find that the selections enriched peptides from proteins that share GO terms related to cellular localization and processes with the baits, supporting that the library do contain potentially relevant ligands, and that the selection enriched for potentially relevant interactors. A similar trend is observed for the proposed phospho-modulated interactions. In terms of reported PPIs, we found that 144 of 884 PPIs found in the study have previous support. We thus find that there is at least some overlap between the domain containing proteins and the phosphosite containing proteins represented in the library.

We have added the information on the shared GO terms to Table S7A and the global analysis of the results to Figure S1F.

Question 3:
I would have liked a better understanding of the library as designed. Specifically, I wished to understand better the following details:

o Given the tiling approach, what is the distribution of positions the phosphosite occupies relative to the ends? Did the HD2 combination with Ochoa selection also steer the library to centralize the phosphosite, or are some or many sites near the end of a peptide?

o How did selection criteria change/alter the sequence distributions of the library? For example, given the phosphoproteome as a whole, the starting HD2 library that has phosphorylation sites, and then the final library that uses the Ochoa constraints - does sequence information remain similarly diverse or does it become increasingly constrained?

Reply:
We agree that this is relevant information that should be added to the manuscript. To answer the questions:

o There is an even distribution of phosphosites along the peptide sequences. We have added this information to Sup Fig 1B. Most phosphosites (90.8%) are covered by 3 or more peptides. This information has also been added to Sup Fig 1A.

o The sequence information (in terms of amino acid distribution) was largely unaffected between the HD2 library design, and the subset included in the phosphomimetic library. After selection, we note an enrichment of tyrosine (2.4-fold more than in the library design) and tryptophan (4 x more than in library design) in the high-to-medium confidence set of ligands. We have added this information to Sup Fig 1G.

Question 4:
A majority of the phosphosites have annotations for kinases, which is a true enrichment, compared to the general phosphoproteome - what is the diversity of kinases connected to these sites? I could see concern for skewing the library towards particular kinases that are well studied/understood and could contribute to a constraint on what the library covered that would be helpful for readers to understand.

Reply:
Indeed, 65% of the phosphosites in the library design are associated with predicted kinases, which in part can be explained by the constraints imposed by the Ochcha et al study. In particular, one (of many) properties used to gain functional confidence in the high-confidence phosphosites was if the sites matched known kinase motifs. Thus, the library might be skewed towards well studied kinases. We have clarified this in the discussion, and we have added a panel showcasing the diversity of predicted kinases to Sup Fig 1 (panel C).

Question 5:
One of the challenges of high throughput binding assays such as this is the protein purification and functional assessment of the domains produced. For example, in the obligate pairs tested with phosphosites, no positive indication of binding was seen for phosphopeptides, suggesting the domain may not have been functional. It would be helpful if the authors could indicate a positive control interaction that was expected for every domain tested and whether it was observed. The lack of observation might suggest which of the domains should be dropped from consideration having demonstrated no function at any point in the experiments. Although the authors make no indication in using or trusting the negative data from this experiment, others in the field may do so and so it could be very helpful to indicate experiments of dubiousness based on domains that gave no positive result.

Reply:
Thank you for the feedback. Indeed, we make no claims related to the negative results, as there are multiple reasons why phage selections may fail to return binders. We agree that it would have been elegant to validate binding of each and every screened domain with previously reported binders, but it is practically not feasible as each binding study requires a fairly large amount of protein and takes about a week of work, including expression and purification of one protein in enough amount amount and with high purity. Moreover, it is not feasible from an economical point of view. Each unlabeled peptide costs about €150-200, and each phosphorylation adds on about €150, and the FITC-label adds on about the same amount. Thus, for validating binding of 70 proteins with 70 positive controls we would need to invest about 1 ½ years time of work to produce proteins, and around €30,000 only in peptide synthesis for control experiments. Thus, although we agree with the reviewer that this would be valuable information, we find that it is not possible to generate this data for the purpose of supporting negative results. That being said, we validated binding of the negative controls Pin1 WW and 14-3-3 theta in the current study. Thus, although not comprehensive, the available results support that the purified proteins are functional.

Question 6:

In the final experiments that take a phosphomodulated motif described into cells this experiment tests the overall interaction (focusing on deleting the 12 amino acids that define the ligand) and not the role for phosphomodulation in regulating the interaction, which seems a major limitation. Given the focus of the method leading up to this it seems more relevant to make point mutations of the phosphosite to phosphomimetic and a non-phosphorylatable amino acid that conserves the motif to truly connect the role of phosphomodulation to the cell cycle and mitosis.

Reply:
This is a valid point. We agree it would have been more precise to test the effect of a point mutation of the phosphosite rather than a truncation of 12 amino acids. We therefore generated a point mutant of HURP (S839A), and showed that this mutation abrogates the cell cycle dependent co-immunoprecipitation of clathrin and HURP. Moreover, we have generated a stable cell line to express mutant HURP, and have validated that the point mutations lead to mitotic delays. We thank the review for the suggestion which helped us to improve the quality of the paper.

Question 7 (Minor comment):
Where peptide bounds are given, it would be helpful to also specify the site of phosphorylation of study in the given target. (e.g. TOM1366-382 which is the phosphosite?)

Reply:
We agree and have added the missing information.

Question 8 (Minor comment):
The authors refer to "promiscuous phosphorylation" in the introduction, which is not a scientific term and (in my opinion) used lazily by scientists to talk about molecular biology that we do not fully understand. I don't see the point of using this term, it detracts from science and in some cultures is offensive. Instead, it would be better to clearly delineate what is meant here - is it truly that there are non-functional phosphorylation sites that are tolerated because they are neutral?

Reply:
We have removed the comment on promiscuous phosphorylation.

We thank review 1 for constructive criticism that helped us improve the quality of the manuscript.

Reviewer #2:

Kliche et al report on a phage display approach with 70 protein domains screening a library of 90000 peptides that cover regions of IDs. These ID regions are known to be post translationally modified/ phosphorylated in patches /cluster (literature is missing). Therefore, the authors include X to E substitutions that resemble 13000 phospho-sites from 6200 proteins in the peptide library. The claim is that this way phospho-modulated motif-based PPIs can be studied on a large scale.
1070 interactions were identified involving 52 domains where 126 PPIs showed a decreased and 126 showed an increased signal with an E substitution. Notably, selections against phospho-serine/threonine-binding domains failed. Individual pairs are tested through displacement binding experiments with synthesized phospho-peptides in use for comparison. I would argue with mixed outcome, which includes low affinities and various diverse effects. For three pairs a motif is scanned with E and pS peptides, again with three different effects. Finally, biological validation of Clathrin binding to a C-terminal HURP peptide motif was found to be cell cycle condition dependent. These experiments are well conducted and assay the peptide motif through deletion of the C-terminal motif. Ser839 is the proposed target for CDKs in the mitotic phase of cells.

Points for consideration

Comment 1:
*) Figure 3 is key to the study.
* Here a volcano plot shows the differences between tiling X to E peptides in binding as determined by sequencing. However, it is clear from the plot that the data analysis is somehow flawed. The P-value is a linear function of the read differences and thus not a measure of statistical significance. I guess this is because of data processing that does not meet high quality criteria.
* First, the cut -off for taking a sequence read into consideration is 1!
* Second, there is no background i.e. sequencing from non selective condition taken into the equation. So enrichment is not quantified.
*Third, all sequences that represent one experiment (i.e. domain) are pooled, so that there are essentially no replicas of the experiment.
*With one value (I guess absolute read count) it is actually very difficult to make any statistical evaluation of the screen.

Reply:
Thank you for the points for consideration. Indeed, figure 3 is a key to study. However, from reading the above points it is clear that the reviewer has misunderstood the experimental data as well as the analysis.

These are the main points that has been misunderstood:

- Although being V-shaped, the axis are not the same as a classic volcano plot and the data should not be interpreted in the same way. To clarify:
  - The y axis is a M-W p-value of all data points for a single mutation across the wt and p-mimetic library
  - The x axis is the sum of the relative differences of each data point for the wt and p-mimetic.
  - The two values are highly correlated as is reflected in the plot
- The goal of the plot is to show these data in a clear spread to allow the most interesting and significant hits to be clearly seen.
- A one dimensional plot of the data from either axis could be used to replace the plot however it is not as easy to highlight multiple labels on such a format

- In relation to the background use, the background used is the NGS count for each respective wildtype peptide as we are aiming to understand the effect of the phosphomimetic mutations in comparison to the wild-type.
- In terms of comparison to the library background, the background is fairly evenly distributed (see Fig 1D and Fig S1E), and the selections clearly enrich for binding peptides.
- Each bait was screened 3 or more times so that every data point includes data from at least 3 replicates
- Given the number of overlapping peptides for each phoshomimetic mutation and the multiple replicates each data point in plot includes information from as many as 15 independent observations

In summary, most of the misunderstanding related to this figure is related to the assumption that figure 3 is a volcano plot and the analysis is similar to an MS analysis. This is not the case. We have tried to clarify the description of the data and the analysis to improve the interpretability of this key plot. Panel A in Fig 1 was also modified to better reflect the fact that a single phosphosite can be covered by multiple overlapping peptides and the distribution of peptides per phosphosite was added to Fig S1 (panel A).

Comment 2:
The study does not convincingly demonstrate that what we are looking at is actually about phosphorylation. Linear Peptide motifs are binding signatures that per definition tell about the influence of amino acid exchanges, including the exchange to E. Any change of amino acid may have an effect (reflected in the motif as such) and for example alter affinities (This is actually concluded in Figure 4A). The potential of how phosphorylation affects motif signature has been studied in a couple of works e.g. by J Reimand et al..

Here it would be important to demonstrate that phosphorylation (as reversible regulatory "mutation") plays a special role in this context, e.g. by frequency, effect size or that phospho-recognition is special.

Reply:
We validated the effect of the phosphorylation as a proxy for serine/threonine phosphorylation by a large number of affinity measurements (see Fig. 3C). Clearly, other modifications in the motifs, or in the flanking regions, may affect binding, but the purpose of this study was not to explore those effects but to develop a protocol for identifying phosphomodulated interactions. While we appreciate the work by Reimand et al. we do not find it relevant for this study.

Comment 3:
However, the data do not support this: phospho-binding domains largely failed

Reply:
As clearly outlined as a limitation of the study in the manuscript, phosphomimetic mutations are not suitable for capturing strictly phospho-dependent interactions.

Comment 4:
Individual cases studied give a set of diverse results (Figure 3B, Figure S4). E.g. GGA1VHS three peptides would represent such a result, where in one case phosphorylation can be important in the other case it is maybe about the mutation. So what if the phospho-peptide has a smaller effect than the mutation and vice versa? Several of the pairs that meet the cut off do not show differences in binding of both the mutation and the phospho-version.

Reply:
We have commented on plausible explanations to these observations in the text, see section 2.4. The mentioned GGA1 VHS case is explained by the low affinity of the interactions which makes the results unreliable.

Comment 5:
What about fully mutagenizing the phospho-sites and see if E/D is actually different than any other mutation (that violates the motif info)?

Reply:
This study is not about exploring the effects of all possible mutations. While it is technically possible to through deep mutational scanning, we find that this is a topic for a different study.

Comment 6
Figure 6: The interaction is clearly depended on synchronizing the cell and nocodazole release. However it is unclear why the control experiments was performed with a C-term deletion and not e.g. with S839A or S839E mutant versions of the proteins. This would much better reflect the approach of the paper and could demonstrate that the non-phosphorylate version S839A is not binding.

Reply:
This is a valid point. We agree and have performed the experiment. We have generated a point mutant of HURP (S839A), and showed that the mutation abrogates the cell cycle dependent co-immunoprecipitation of clathrin and HURP. Moreover, we have generated a stable cell line to express mutant HURP, and have validated that the point mutations lead to mitotic delays.

Reviewer #3:

Kliche et al. performed a large-scale study to identify phospho-regulated protein-protein interactions using their previously established proteomic peptide-phage display (Prop-PD) method. Overall, they screened 73 modular domains and identified 252 putative phosphor-modulated interactions. They further performed affinity analyses on a subset of 21 interactions. Finally, they studied a novel phospho-dependent interaction between clathrin and the mitotic spindle protein HURP in detail using various biophysical, molecular assays and analyses.

Comment 1:
My main criticism is that the current study seems to be a scale-up from the authors' previous small-scale study from 2018 in the same journal (Sundell et al. Mol Syst Biol) to identify phosho-regulated interactions for PDZ domains.

Reply:
While we appreciate the opinion of the reviewer we respectfully disagree. We find it a strength, not a weakness, of our research that we systematically build upon previous results and expand and develop methods and scopes. The pilot study was only focused on Ser/Thr phosphosites in the C-terminal regions of the human proteome, and the phosphomodulation of interactions with a select set of PDZ domains. Here, we expand the approach to phosphosites in the disordered regions of the human proteome, and test the approach against over 70 different domains. Thus, the scope of the approach is massively expanded and we now have a broadly applicable resource that will be highly valuable for future studies. We have clarified this advancement in the introduction and in the discussion.

In addition to the method development, we performed in depth structural and functional validation of a select case, in particular the phosphomodulation of the cell cycle dependent HURP-clathrin interaction. Thus, while we appreciate that the reviewer acknowledges our previous work, we find that our study represents an important contribution to the field.

Comment 2:
I recommend the authors to develop a user-friendly webserver to the present their data so that other researchers in the field can tactfully explore their results.

Reply:
We agree and we have developed a webserver for all our interaction data (http://slim.icr.ac.uk/proppd/). We are pleased to release the database to the public together with this manuscript. The website contains the results of this study, together with other released studies, and will be updated in connection to future date releases.

Comment 3:
Can the authors comment on their understanding of the role of Histidine phosphorylation in cellular functions? Can they also discuss if they made any efforts to study the role of Histidine phosphorylation in regulating protein-protein interactions?

Reply:
We acknowledge that the topic of histidine phosphorylation is interesting, and we are aware of the recent studies in the field (e.g. Luthala et al, on biorxiv.. and the review by Hunter (2022). We are also aware that some studies (e.g Leijten et al., (2020) have questioned the importance of histidine phosphorylation in mammalian signaling. As we are not experts on histidine phosphorylation, and it is not the topic of the study we refrain from discussing the importance of histidine phosphorylation in the manuscript. However, we now mention this modification in the introduction and the discussion. To satisfy the curiosity of the reviewer we can disclose that we so far did not make any efforts to study histidine phosphorylation.

As histidine phosphorylation is not the topic of this study, we are surprised that this is raised as a major concern, and cannot take this comment as a serious criticism of our study.

Comment 4 (minor):
What was the criteria for selecting 32 interactions for the affinity analysis?

Reply:
Interactions for validations were selected to cover a broad panel of domains, different motifs, and a broad range of phosphomimetic enrichment scores.

Comment 5 (minor):

The authors need to discuss potential limitations of their approach in understanding phosphor-mediated protein-protein interactions.

Reply:
As indicated by reviewer 1 we have clearly discussed the limitations of the approach. We mention it in the result section, and have now further elaborated on the limitations in the discussion.

Added experiments:

During the revision of our study, we became interested in the possible competition between GTSE1 (G2 and S phase expressed-protein 1) and HURP for clathrin binding, which we investigated on the peptide level. GTSE1 has been reported to bind to the clathrin NTD by multiple LIxF motifs and the interaction to be important for microtubule stabilisation during mitosis (Rondelet et al., 2020). This places GTSE1, HURP and clathrin in the same biological context. Overlaying of the clathrin NTD structures either complexed with HURP or GTSE1 peptides revealed that both peptides can be accommodated by the same binding pockets on clathrin. We show that a competition between GTSE1 and HURP for clathrin is possible on the peptide level, and that their interactions do not adhere to the same phospho-modulation. We have added this information to the manuscript during the revision.

22nd Mar 2023

Manuscript Number: MSB-2022-11164R-Q
Title: Large-scale identification of phospho-modulated motif-based protein-protein interactions

Dear Ylva,

Thank you again for submitting your work to Molecular Systems Biology. We have now heard back from the two reviewers (previous reviewers #1 and #2) who were asked to evaluate your study. As you will see below, the reviewers think that the study has improved as a result of the performed revisions and they support publication. Reviewer #2 lists a few remaining concerns, which we would ask you to address in a final round of revision by providing some further clarifications and performing some text edits.

We would also ask you to address some editorial issues listed below.

- Please provide a .doc version of the manuscript text (including legends for main figures) and individual production quality figure files for the main Figures (one file per figure).

- We have replaced Supplementary Information by the Expanded View (EV format). In this case, all additional figures can be included in a PDF called Appendix. Appendix figures should be labeled and called out as: "Appendix Figure S1, Appendix Figure S2..." etc. Each legend should be below the corresponding Figure in the Appendix. Please include a Table of Contents in the beginning of the Appendix. For detailed instructions regarding expanded view please refer to our Author Guidelines: .

- Tables S1-S12 should be provided as Datasets EV1-EV12. Please provide one file per Dataset. Each file should contain a brief description of the Dataset in a separate tab of the .xls file.

- Please include 5 keywords.

- Please provide a "standfirst text" summarizing the study in one or two sentences (approximately 250 characters), three to four "bullet points" highlighting the main findings and a "synopsis image" (550px width and max 400px height, jpeg format) to highlight the paper on our homepage.

- All Materials and Methods need to be described in the main text. We would encourage you to use 'Structured Methods', our new Materials and Methods format. According to this format, the Material and Methods section should include a Reagents and Tools Table (listing key reagents, experimental models, software and relevant equipment and including their sources and relevant identifiers) followed by a Methods and Protocols section in which we encourage the authors to describe their methods using a step-by-step protocol format with bullet points, to facilitate the adoption of the methodologies across labs. More information on how to adhere to this format as well as downloadable templates (.doc or .xls) for the Reagents and Tools Table can be found in our author guidelines: . An example of a Method paper with Structured Methods can be found here:

- Please include a "Disclosure & Competing Interests Statement" in the main text.

- For data quantification: please specify the name of the statistical test used to generate error bars and P values, the number (n) of independent experiments (specify technical or biological replicates) underlying each data point and the test used to calculate p-values in each figure legend. The figure legends should contain a basic description of n, P and the test applied. Graphs must include a description of the bars and the error bars (s.d., s.e.m.).

- Please include a Data availability section describing how the data generated in the study has been made available. Datasets and code generated in the study should be listed in a structured manner in the "Data Availability" section (after the Materials & Methods). If your study does not include datasets, please insert the following statement: This study includes no data deposited in external repositories.

- Please format the References according to the Molecular Systems Biology reference style i.e. ordered alphabetically and listing the first 10 authors followed by et al.

- When you resubmit your manuscript, please download our CHECKLIST (https://bit.ly/EMBOPressAuthorChecklist) and include the completed form in your submission. *Please note* that the Author Checklist will be published alongside the paper as part of the transparent process (https://www.embopress.org/page/journal/17444292/authorguide#transparentprocess)

Please resubmit your revised manuscript online, with a covering letter listing amendments and responses to each point raised by the referees. Please resubmit the paper **within one month** and ideally as soon as possible. If we do not receive the revised

manuscript within this time period, the file might be closed and any subsequent resubmission would be treated as a new manuscript. Please use the Manuscript Number (above) in all correspondence.

Click on the link below to submit your revised paper.

https://msb.msubmit.net/cgi-bin/main.plex

As a matter of course, please make sure that you have correctly followed the instructions for authors as given on the submission website.

Thank you for submitting this paper to Molecular Systems Biology.

Best wishes,

Maria

Maria Polychronidou, PhD
Senior Editor
Molecular Systems Biology

If you do choose to resubmit, please click on the link below to submit the revision online before 21st Apr 2023.

https://msb.msubmit.net/cgi-bin/main.plex

IMPORTANT: When you send your revision, we will require the following items:
1. the manuscript text in LaTeX, RTF or MS Word format
2. a letter with a detailed description of the changes made in response to the referees. Please specify clearly the exact places in the text (pages and paragraphs) where each change has been made in response to each specific comment given
3. three to four 'bullet points' highlighting the main findings of your study
4. a short 'blurb' text summarizing in two sentences the study (max. 250 characters)
5. a 'thumbnail image' (550px width and max 400px height, Illustrator, PowerPoint or jpeg format), which can be used as 'visual title' for the synopsis section of your paper.
6. Please include an author contributions statement after the Acknowledgements section (see https://www.embopress.org/page/journal/17444292/authorguide#manuscriptpreparation)
7. Please complete the CHECKLIST available at (https://bit.ly/EMBOPressAuthorChecklist).
Please note that the Author Checklist will be published alongside the paper as part of the transparent process (https://www.embopress.org/page/journal/17444292/authorguide#transparentprocess).
8. When assembling figures, please refer to our figure preparation guideline in order to ensure proper formatting and readability in print as well as on screen:
https://bit.ly/EMBOPressFigurePreparationGuideline
See also figure legend guidelines: https://www.embopress.org/page/journal/17444292/authorguide#figureformat
9. Please note that corresponding authors are required to supply an ORCID ID for their name upon submission of a revised manuscript (EMBO Press signed a joint statement to encourage ORCID adoption).
(https://www.embopress.org/page/journal/17444292/authorguide#editorialprocess)
Currently, our records indicate that the ORCID for your account is 0000-0002-7081-3846.

Please click the link below to modify this ORCID:
Link Not Available

The system will prompt you to fill in your funding and payment information. This will allow Wiley to send you a quote for the article processing charge (APC) in case of acceptance. This quote takes into account any reduction or fee waivers that you may be eligible for. Authors do not need to pay any fees before their manuscript is accepted and transferred to the publisher.

As a matter of course, please make sure that you have correctly followed the instructions for authors as given on the submission website.

*** PLEASE NOTE *** As part of the EMBO Press transparent editorial process initiative (see our Editorial at https://dx.doi.org/10.1038/msb.2010.72 , Molecular Systems Biology will publish online a Review Process File to accompany accepted manuscripts. When preparing your letter of response, please be aware that in the event of acceptance, your cover letter/point-by-point document will be included as part of this File, which will be available to the scientific community. More

information about this initiative is available in our Instructions to Authors. If you have any questions about this initiative, please contact the editorial office (msb@embo.org).

Reviewer #1:

The authors were very thorough in incorporating feedback and/or giving rational considerations for places where incorporation was not possible. I think this paper will be broadly interesting to a range of scientists and sets an approach to the feasibility of approaching large scale phospho-mediated interactions. I still greatly appreciate the authors are very transparent about the limitations and have confirmatory evidence for examples.

Reviewer #2:

Review on "Large-scale identification of phospho-modulated motif-based protein-protein

The authors resubmit with improvements apparently addressing the comments of the other reviewers. With the comments from my side, I get the impression that they were deemed wrong, which can well be the case. However, except for the validation (clathrin PPI), the authors showed no significant attempts to explain why this is the case, neither in the rebuttal nor in the manuscript.

Screen:
52 domains are scanned against a peptide library that tiles disordered regions using 16 amino acid peptides, plus the corresponding tiles with ~13,500 serine/threonine phosphomimetic S/T to E mutations.
This means we do not know whether the motif is phospho-sensitive or whether we are looking at a mutation at the position. As there is no background that can distinguish the two alternatives, we are left with a somewhat naive comparison of S/T versus E. In my previous comments I was suggesting (more as a theoretical question, and to illustrate my point) to use other mutations S/T to X only on the predicted S/T to see if e.g. S/T to X would give similar enrichment for interaction perturbation (a baseline of effects). Given the experimental setup it is possible that any mutation of S/T will change the interaction to a similar extend. I think this scenario should be discussed (again: Figure 4 shows this as a conclusion: mutation of consensus motif vs phosphorylation). This is does not have a lot to do with the fact that that phospho-mimicry mutations did not work for strictly phospho-binding domains.

Validation:
In the validation experiments that deal with individual cases, the PPIs are well characterized. However, based on 32 pairings (23 peptide pairs) we see in Figure 3B that overall the validation does show differences to the screening, both grey and opposite colors in various combinations. How does 3B exactly relate to Figure F3 (legends of the figures do not match and it says "representative peptide pairs based on the PM_HD2 selection data ")? For the correlation with PES values, the authors report the success rates in the text clearly: disabling (5 out of 7 confirmed); enabling (9 out of 11 confirmed); (4 out of 18) of the cases failed. However, which pairs are shown in figure 3F?
The validation works on a case by case and there is no good idea how to generally assess the data ending up in the discussion by saying 77% correctly identified interactions. The overarching question here is how do we extrapolate (from the data in figure 3) and assess the meaning of the 250 phospho-site mutations that showed differences to wild type?

Data processing:
I understand now that the variation and thus the stats stem from overlapping peptides and not from multiple measurements (better explained in the text). Since differences of read counts (normalized)are taken and the median read count is 10 only (fig S1 E), it is unclear how the data would look like with a different cutoff. A simple question with regards to PES: How many one peptide differences, two peptide differences, three peptide difference , etc ... are contributing to the significant site - domain pairs.

Summary:
The screen provides a ~250 peptide-domain set where S/T to E mutation impacts binding. A selected set of these interactions is well characterized biophysically and a well worked out biological examples is given.

For the whole data set, I would argue that phospho-modulation is somewhat an overstatement. In the text phosphorylation is sometimes used synonymously with mutation to E.
Overall the paper including the title is not always exact when it speaks about phosphorylation and when about a mutation. With regards to the title: It is something like "large scale identification of mimicry mutations of phospho-sites altering peptide-domain interactions"
Another example: it should say on page 6 top paragraph "The remainder of our study is thus focused on cases in which ... mutation of S/T to E ... abolished or modulated the affinity of interactions. (not phosphorylation).

My suggestion

is - if comments of other reviewers allow - to go ahead and accept the manuscript. Many aspects of the paper will be of large interest to the community and the interaction studies are performed on a very high experimental level including the validation of Clathrin NTD binding to phospho- HURP C-terminus.

Reply to reviewer comments

Reviewer #1:

The authors were very thorough in incorporating feedback and/or giving rational considerations for places where incorporation was not possible. I think this paper will be broadly interesting to a range of scientists and sets an approach to the feasibility of approaching large scale phospho-mediated interactions. I still greatly appreciate the authors are very transparent about the limitations and have confirmatory evidence for examples.

Reply

We thank reviewer #1 for appreciating our study.

Reviewer #2:

Review on "Large-scale identification of phospho-modulated motif-based protein-protein

The authors resubmit with improvements apparently addressing the comments of the other reviewers. With the comments from my side, I get the impression that they were deemed wrong, which can well be the case. However, except for the validation (clathrin PPI), the authors showed no significant attempts to explain why this is the case, neither in the rebuttal nor in the manuscript.

Screen:
52 domains are scanned against a peptide library that tiles disordered regions using 16 amino acid peptides, plus the corresponding tiles with ~13,500 serine/threonine phosphomimetic S/T to E mutations.

This means we do not know whether the motif is phospho-sensitive or whether we are looking at a mutation at the position. As there is no background that can distinguish the two alternatives, we are left with a somewhat naive comparison of S/T versus E. In my previous comments I was suggesting (more as a theoretical question, and to illustrate my point) to use other mutations S/T to X only on the predicted S/T to see if e.g. S/T to X would give similar enrichment for interaction perturbation (a baseline of effects). Given the experimental setup it is possible that any mutation of S/T will change the interaction to a similar extend. I think this scenario should be discussed (again: Figure 4 shows this as a conclusion: mutation of consensus motif vs phosphorylation). This is does not have a lot to do with the fact that that phospho-mimicry mutations did not work for strictly phospho-binding domains.

Reply

It is a well-known fact that affinities are affected by perturbations of binding sites such as mutations and PTMs. We agree that phosphorylation, phosphomimetic mutations as well as other mutations may perturb interactions in our set up as well as in a cellular context. We do not see this as something specific for the method, but as a general fact related to molecular recognition. In this study we were not aiming to explore the generality of how binding is affected by different kinds of perturbations, but we specifically asked if we would use phosphomimetic as a proxy for Ser/Thr phosphorylation to identify phospho-modulated interactions. For this reason, we took great care in designing the library, only mutating positions that are reported to be subjected to phosphorylation. Thus, we decided not to indulge in an extended discussion on the many ways interactions may be modulated by other mutations (or other PTMs), and we hope that this is fine given the scope of the manuscript. Nevertheless, we have added the following sentence to the discussion

"For cases when the results suggest reduced affinity upon phosphomimetic mutations of core motif residues, other mutations (e.g. disease related mutations) may similarly perturb binding."

Validation:
In the validation experiments that deal with individual cases, the PPIs are well characterized. However, based on 32 pairings (23 peptide pairs) we see in Figure 3B that overall the validation does show differences to the screening, both grey and opposite colors in various combinations.

How does 3B exactly relate to Figure F3 (legends of the figures do not match and it says "representative peptide pairs based on the PM_HD2 selection data ")?
For the correlation with PES values, the authors report the success rates in the text clearly: disabling (5 out of 7 confirmed); enabling (9 out of 11 confirmed); (4 out of 18) of the cases failed. However, which pairs are shown in figure 3F?

Reply
We thank the reviewer for finding a mistake in the figure legend caused by shifting the position of the panels. It has been corrected so that Figure 3E and F have their corresponding descriptions. To answer the question:

- *Figure 3B represents the peptides and bait protein tested in affinity measurements.*
- *The coloring of the gene names of the prey proteins corresponds to the coloring in Figure 3A, i.e. to the binding preferences established by the phage display. Essentially:*
  - *blue = wild-type preferred in the phage display*
  - *grey = no preference for either*
  - *orange = phosphomimetic preferred in the phage display.*
- *The coloring of the names also corresponds to Figure 3F, with the exception that the orange (phosphomimetics) has been replaced for red (phospho).*
- *The affinities of the phospho-peptides have been used for plotting Figure 3F, and the numbers in the text correspond to the data shown in Figure 3F.*

We understand that the differential color-coding between 3B and 3F when it comes to the fold-change in affinity might be confusing. We have therefore removed the fold-change affinity color axes in Figure 3B. These are still represented by Table 1 and the y-axes in Figure 3F.

Validation
The validation works on a case by case and there is no good idea how to generally assess the data ending up in the discussion by saying 77% correctly identified interactions. The overarching question here is how do we extrapolate (from the data in figure 3) and assess the meaning of the 250 phospho-site mutations that showed differences to wild type?

Reply
Like results from any large-scale analysis the results should be handled with care. We found 895 domain-peptide interactions involving 54 bait protein domains and 537 peptide-containing proteins. Our results suggest that 248 (22%) of the phospho-sites found in these peptides modulate the affinity of the interactions. Through our extensive low-throughput validations (and yes, I would say they are extensive given the cost and time required for these dedicated experiments) we found that the outcome of phosphomimetic ProP-PD correctly predicts the effect of Ser/Thr phosphorylation on binding in 78% of cases. Thus, we would expect the user of the data to i) use the protein-peptide interaction data in a sensible manner, understanding that a higher confidence means higher probability of an interaction to be biophysically true, and ii) understand that the accuracy of the probability of the suggested

effect on binding by phosphorylation is in the order of 80%, a number which is likely higher if the phosphosites are overlapping with the consensus motifs for the baits used. We find that these points are already explained extensively in the manuscript.

Data processing:
I understand now that the variation and thus the stats stem from overlapping peptides and not from multiple measurements (better explained in the text). Since differences of read counts (normalized) are taken and the median read count is 10 only (fig S1 E), it is unclear how the data would look like with a different cutoff. A simple question with regards to PES: How many one peptide differences, two peptide differences, three peptide difference , etc ... are contributing to the significant site - domain pairs.

Reply
The distribution shown in Fig S1 E is related to the coverage of the naive library counts, not the selection results and the counts cut-off was systematically established in a previous publication (Benz et al. 2022). But regarding the question, the raw read count distances among all binding enriched data and also the ranked data that supports our classification of phosphosites as "putative phosphomodulated" cover a wide range including low values (1, 2, 3, etc) for both groups. But the distribution of these distances increases in the putative phosphomodulated group, as evidenced by the increase on the median and other percentile values (see included figure and table below).

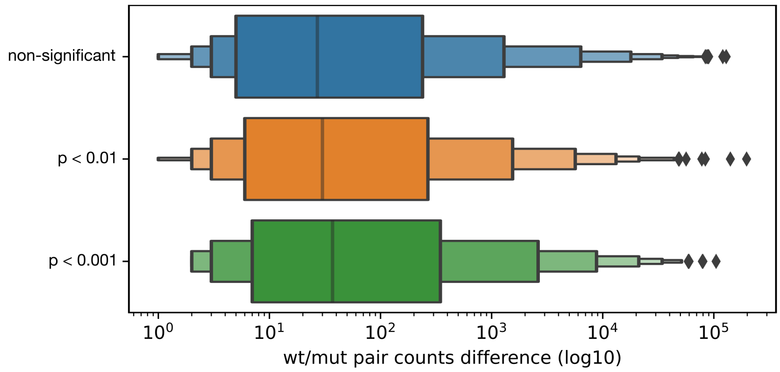

	N	Range	Mean	percentiles values 25%	50%	75%
**non-significant**	3153	1-129185	1863.6	5	27	239
**p<0.01**	1573	1-197188	2062.7	6	33	292
**p<0.001**	712	2-104793	2320.2	7	37	345

Summary:
The screen provides a ~250 peptide-domain set where S/T to E mutation impacts binding. A selected set of these interactions is well characterized biophysically and a well worked out biological examples is given.

For the whole data set, I would argue that phospho-modulation is somewhat an overstatement. In the text phosphorylation is sometimes used synonymously with mutation to E. Overall the paper including the title is not always exact when it speaks about phosphorylation and when about a mutation. With regards to the title: It is something like "large scale identification of mimicry mutations of phospho-sites altering peptide-domain interactions"

Another example: it should say on page 6 top paragraph "The remainder of our study is thus focused on cases in which ... mutation of S/T to E ... abolished or modulated the affinity of interactions. (not phosphorylation).

***Reply*:**
Following the reviewer's suggestions we have updated the title to more correctly reflect the content of the paper. It now reads:
"Large-scale phosphomimetic screening identifies phospho-modulated motif-based protein interactions".

We have modified the statement to "The remainder of our study is thus focused on cases in which phospho-mimetic mutations and phosphorylation abolished or modulated the affinity of interactions".

My suggestion
is - if comments of other reviewers allow - to go ahead and accept the manuscript. Many aspects of the paper will be of large interest to the community and the interaction studies are performed on a very high experimental level including the validation of Clathrin NTD binding to phospho- HURP C-terminus.

Reply:
We thank the reviewer for the time and effort invested in reviewing the manuscript, and for the comments and suggestions that helped us to improve the manuscript.

28th Apr 2023

Manuscript Number: MSB-2022-11164RR
Title: Large-scale phosphomimetic screening identifies phospho-modulated motif-based protein interactions

Dear Ylva,

Thank you for sending us your revised manuscript. We think that the remaining concerns of reviewer #2 have been satisfactorily addressed. I am glad to inform you that we can soon accept your study for publication, pending some minor remaining issues listed below.

- Our data editors and I have made some minor formatting changes (see attached file). Please make all requested text changes using the attached file and *keeping the "track changes" mode* so that we can easily access the edits made.

- In the main text: the callout for Fig. 3B should be after the callout for Fig. 3A.

- Please include callouts for Fig. 6C, Appendix Fig. S4, Appendix Fig. 54A-D, Dataset EV9.

- Please include page numbers in the Appendix Table of Contents.

Please resubmit your revised manuscript online, **within two weeks** and ideally as soon as possible. If we do not receive the revised manuscript within this time period, the file might be closed and any subsequent resubmission would be treated as a new manuscript. Please use the Manuscript Number (above) in all correspondence.

Click on the link below to submit your revised paper.

https://msb.msubmit.net/cgi-bin/main.plex

Thank you for submitting this paper to Molecular Systems Biology.

Best wishes,

Maria

Maria Polychronidou, PhD
Senior Editor
Molecular Systems Biology

If you do choose to resubmit, please click on the link below to submit the revision online before 14th May 2023.

https://msb.msubmit.net/cgi-bin/main.plex

IMPORTANT: Please note that corresponding authors are required to supply an ORCID ID for their name upon submission of a revised manuscript (EMBO Press signed a joint statement to encourage ORCID adoption).
(https://www.embopress.org/page/journal/17444292/authorguide#editorialprocess)
Currently, our records indicate that the ORCID for your account is 0000-0002-7081-3846.

Please click the link below to modify this ORCID:
Link Not Available

The system will prompt you to fill in your funding and payment information. This will allow Wiley to send you a quote for the article processing charge (APC) in case of acceptance. This quote takes into account any reduction or fee waivers that you may be eligible for. Authors do not need to pay any fees before their manuscript is accepted and transferred to the publisher.

*** PLEASE NOTE *** As part of the EMBO Press transparent editorial process initiative (see our Editorial at https://dx.doi.org/10.1038/msb.2010.72 , Molecular Systems Biology will publish online a Review Process File to accompany accepted manuscripts. When preparing your letter of response, please be aware that in the event of acceptance, your cover letter/point-by-point document will be included as part of this File, which will be available to the scientific community. More

information about this initiative is available in our Instructions to Authors. If you have any questions about this initiative, please contact the editorial office (msb@embo.org).

All editorial and formatting issues were resolved by the authors.

3rd May 2023

Manuscript number: MSB-2022-11164RRR
Title: Large-scale phosphomimetic screening identifies phospho-modulated motif-based protein interactions

Dear Ylva,

Thank you again for sending us your revised manuscript. We are now satisfied with the modifications made and I am pleased to inform you that your paper has been accepted for publication.

*** PLEASE NOTE *** As part of the EMBO Publications transparent editorial process initiative (see our Editorial at https://dx.doi.org/10.103/msb.2010.72), Molecular Systems Biology publishes online a Review Process File with each accepted manuscripts. This file will be published in conjunction with your paper and will include the anonymous referee reports, your point- by-point response and all pertinent correspondence relating to the manuscript. If you do NOT want this File to be published, please inform the editorial office at msb@embo.org within 14 days upon receipt of the present letter.

LICENSE AND PAYMENT:
All articles published in Molecular Systems Biology are fully open access: immediately and freely available to read, download and share.

Molecular Systems Biology charges an article processing charge (APC) to cover the publication costs. You, as the corresponding author for this manuscript, should have already received a quote with the article processing fee separately. Please let us know in case this quote has not been received.

Once your article is at Wiley for editorial production, you will receive an email from Wiley's Author Services system, which will ask you to log in and will present you with the publication license form for completion. Within the same system the publication fee can be paid by credit card, an invoice or pro forma can be requested.

Payment of the publication charge and the signed Open Access Agreement form must be received before the article can be published online.

Upon acceptance it is mandatory for you to return the completed payment form. Failure to send back the form may result in a delay in the publication of your paper.

Molecular Systems Biology articles are published under the Creative Commons licence CC BY, which facilitates the sharing of scientific information by reducing legal barriers, while mandating attribution of the source in accordance to standard scholarly practice.

Proofs will be forwarded to you within the next 2-3 weeks.

Thank you very much for submitting your work to Molecular Systems Biology.

Kind regards,

Maria

Maria Polychronidou, PhD
Senior Editor
Molecular Systems Biology

EMBO Press Author Checklist

Corresponding Author Name: Ylva Ivarsson
Journal Submitted to: Molecular Systems Biology
Manuscript Number: MSB-2022-11164R-Q

USEFUL LINKS FOR COMPLETING THIS FORM
The EMBO Journal - Author Guidelines
EMBO Reports - Author Guidelines
Molecular Systems Biology - Author Guidelines
EMBO Molecular Medicine - Author Guidelines

Reporting Checklist for Life Science Articles (updated January
This checklist is adapted from Materials Design Analysis Reporting (MDAR) Checklist for Authors. MDAR establishes a minimum set of requirements in transparent reporting in the life sciences (see Statement of Task: 10.31222/osf.io/9sm4x). Please follow the journal's guidelines in preparing your
Please note that a copy of this checklist will be published alongside your article.

Abridged guidelines for figures
1. Data
The data shown in figures should satisfy the following conditions:
- the data were obtained and processed according to the field's best practice and are presented to reflect the results of the experiments in an accurate and unbiased manner.
- ideally, figure panels should include only measurements that are directly comparable to each other and obtained with the same assay.
- plots include clearly labeled error bars for independent experiments and sample sizes. Unless justified, error bars should not be shown for technical
- if n<5, the individual data points from each experiment should be plotted. Any statistical test employed should be justified.
- Source Data should be included to report the data underlying figures according to the guidelines set out in the authorship guidelines on Data

2. Captions
Each figure caption should contain the following information, for each panel where they are relevant:
- a specification of the experimental system investigated (eg cell line, species name).
- the assay(s) and method(s) used to carry out the reported observations and measurements.
- an explicit mention of the biological and chemical entity(ies) that are being measured.
- an explicit mention of the biological and chemical entity(ies) that are altered/varied/perturbed in a controlled manner.
- the exact sample size (n) for each experimental group/condition, given as a number, not a range;
- a description of the sample collection allowing the reader to understand whether the samples represent technical or biological replicates (including how many animals, litters, cultures, etc.).
- a statement of how many times the experiment shown was independently replicated in the laboratory.
- definitions of statistical methods and measures:
  - common tests, such as t-test (please specify whether paired vs. unpaired), simple χ^2 tests, Wilcoxon and Mann-Whitney tests, can be unambiguously identified by name only, but more complex techniques should be described in the methods section;
  - are tests one-sided or two-sided?
  - are there adjustments for multiple comparisons?
  - exact statistical test results, e.g., P values = x but not P values < x;
  - definition of 'center values' as median or average;
  - definition of error bars as s.d. or s.e.m.

Please complete ALL of the questions below.
Select "Not Applicable" only when the requested information is not relevant for your study.

Materials

Newly Created Materials	Information included in the manuscript?	In which section is the information available? (Reagents and Tools Table, Materials and Methods, Figures, Data Availability Section)
New materials and reagents need to be available; do any restrictions apply?	Yes	Reagents & tools table. Restriction: the phage library as the phagemid vector was obtained under a MTA.

Antibodies	Information included in the manuscript?	In which section is the information available? (Reagents and Tools Table, Materials and Methods, Figures, Data Availability Section)
For **antibodies** provide the following information:<br>- Commercial antibodies: RRID (if possible) or supplier name, catalogue number and or/clone number<br>- Non-commercial: RRID or citation	Yes	Reagents & tools table, except one in house ab

DNA and RNA sequences	Information included in the manuscript?	In which section is the information available? (Reagents and Tools Table, Materials and Methods, Figures, Data Availability Section)
**Short novel DNA or RNA including primers, probes**: provide the sequences.	Yes	Reagents and Tools table

Cell materials	Information included in the manuscript?	In which section is the information available? (Reagents and Tools Table, Materials and Methods, Figures, Data Availability Section)
**Cell lines:** Provide species information, strain. Provide accession number in repository **OR** supplier name, catalog number, clone number, and/**OR** RRID.	Yes	See Reagents and Tools table
**Primary cultures:** Provide species, strain, sex of origin, genetic modification status.	Not Applicable	
Report if the cell lines were recently **authenticated** (e.g., by STR profiling) and tested for mycoplasma contamination.	Not Applicable	Cell lines were not recently authenticated and they were not tested for mycoplasma

Experimental animals	Information included in the manuscript?	In which section is the information available? (Reagents and Tools Table, Materials and Methods, Figures, Data Availability Section)
**Laboratory animals or Model organisms:** Provide species, strain, sex, age, genetic modification status. Provide accession number in repository **OR** supplier name, catalog number, clone number, **OR** RRID.	Not Applicable	
**Animal observed in or captured from the field:** Provide species, sex, and age where possible.	Not Applicable	
Please detail **housing and husbandry conditions**.	Not Applicable	

Plants and microbes	Information included in the manuscript?	In which section is the information available? (Reagents and Tools Table, Materials and Methods, Figures, Data Availability Section)
**Plants:** provide species and strain, ecotype and cultivar where relevant, unique accession number if available, and source (including location for collected wild specimens).	Not Applicable	
**Microbes:** provide species and strain, unique accession number if available, and source.	Yes	Reagents & tools table (Source & catalogue number)

Human research participants	Information included in the manuscript?	In which section is the information available? (Reagents and Tools Table, Materials and Methods, Figures, Data Availability Section)
If collected and within the bounds of privacy constraints report on age, sex and gender or ethnicity for all study participants.	Not Applicable	

Core facilities	Information included in the manuscript?	In which section is the information available? (Reagents and Tools Table, Materials and Methods, Figures, Data Availability Section)
If your work benefited from core facilities, was their service mentioned in the acknowledgments section?	Yes	Reagents & tools table: NGS-NGI SciLifeLab facility. Also mentioned in acknowledgment.

Design

Study protocol	Information included in the manuscript?	In which section is the information available? (Reagents and Tools Table, Materials and Methods, Figures, Data Availability Section)
If study protocol has been **pre-registered, provide DOI in the manuscript**. For clinical trials, provide the trial registration number **OR** cite DOI.	Not Applicable	
Report the **clinical trial registration number** (at ClinicalTrials.gov or equivalent), where applicable.	Not Applicable	

Laboratory protocol	Information included in the manuscript?	In which section is the information available? (Reagents and Tools Table, Materials and Methods, Figures, Data Availability Section)
Provide DOI OR other citation details if **external detailed step-by-step protocols** are available.	Not Applicable	

Experimental study design and statistics	Information included in the manuscript?	In which section is the information available? (Reagents and Tools Table, Materials and Methods, Figures, Data Availability Section)
Include a statement about **sample size** estimate even if no statistical methods were used.	Not Applicable	
Were any steps taken to minimize the effects of subjective bias when allocating animals/samples to treatment (e.g. **randomization procedure**)? If yes, have they been described?	Not Applicable	
Include a statement about **blinding** even if no blinding was done.	Not Applicable	
Describe **inclusion/exclusion criteria** if samples or animals were excluded from the analysis. Were the criteria pre-established?<br><br>If sample or data points were omitted from analysis, report if this was due to attrition or intentional exclusion and provide justification.	Yes	Method section: FP measurement. Omitting data due to FITC-contamination of unlabelled peptides. Also indicated in the extended view of the FP experiments.
For every figure, are **statistical tests** justified as appropriate? Do the data meet the assumptions of the tests (e.g., normal distribution)? Describe any methods used to assess it. Is there an estimate of variation within each group of data? Is the variance similar between the groups that are being statistically compared?	Yes	Figure legends and methods.

Sample definition and in-laboratory replication	Information included in the manuscript?	In which section is the information available? (Reagents and Tools Table, Materials and Methods, Figures, Data Availability Section)
In the figure legends: state number of times the experiment was **replicated** in laboratory.	Yes	Figure legends
In the figure legends: define whether data describe **technical or biological replicates**.	Yes	Figure legends

Ethics

Ethics	Information included in the manuscript?	In which section is the information available? (Reagents and Tools Table, Materials and Methods, Figures, Data Availability Section)
Studies involving **human participants**: State details of **authority granting ethics approval** (IRB or equivalent committee(s), provide reference number for approval.	Not Applicable	
Studies involving **human participants**: Include a statement confirming that **informed consent** was obtained from all subjects and that the experiments conformed to the principles set out in the WMA Declaration of Helsinki and the Department of Health and Human Services Belmont Report.	Not Applicable	
Studies involving **human participants:** For publication of **patient photos**, include a statement confirming that consent to publish was obtained.	Not Applicable	
Studies involving experimental **animals**: State details of **authority granting ethics approval** (IRB or equivalent committee(s), provide reference number for approval. Include a statement of compliance with ethical regulations.	Not Applicable	
Studies involving **specimen and field samples:** State if relevant **permits** obtained, provide details of authority approving study; if none were required, explain why.	Not Applicable	

Dual Use Research of Concern (DURC)	Information included in the manuscript?	In which section is the information available? (Reagents and Tools Table, Materials and Methods, Figures, Data Availability Section)
Could your study fall under dual use research restrictions? Please check biosecurity documents and list of **select agents and toxins** (CDC): https://www.selectagents.gov/sat/list.htm	Not Applicable	
If you used a select agent, is the security level of the lab appropriate and reported in the manuscript?	Not Applicable	
If a study is subject to dual use research of concern regulations, is the name of the **authority granting approval and reference number** for the regulatory approval provided in the manuscript?	Not Applicable	

Reporting

The MDAR framework recommends adoption of discipline-specific guidelines, established and endorsed through community initiatives. Journals have their own policy about requiring specific guidelines and recommendations to complement MDAR.

Adherence to community standards	Information included in the manuscript?	In which section is the information available? (Reagents and Tools Table, Materials and Methods, Figures, Data Availability Section)
State if relevant guidelines or checklists (e.g., **ICMJE, MIBBI, ARRIVE, PRISMA**) have been followed or provided.	Not Applicable	
For **tumor marker prognostic studies,** we recommend that you follow the **REMARK** reporting guidelines (see link list at top right). See author guidelines, under 'Reporting Guidelines'. Please confirm you have followed these guidelines.	Not Applicable	
For **phase II and III randomized controlled trials**, please refer to the **CONSORT** flow diagram (see link list at top right) and submit the CONSORT checklist (see link list at top right) with your submission. See author guidelines, under 'Reporting Guidelines'. Please confirm you have submitted this list.	Not Applicable	

Data Availability

Data availability	Information included in the manuscript?	In which section is the information available? (Reagents and Tools Table, Materials and Methods, Figures, Data Availability Section)
Have **primary datasets** been deposited according to the journal's guidelines (see 'Data Deposition' section) and the respective accession numbers provided in the Data Availability Section?	Yes	Interaction data has been deposited in IntAct with the identifier IM-29716. The crystal structure has been deposited in PDB and is available with the PDB ID: 7ZX4
Were **human clinical and genomic datasets** deposited in a public access-controlled repository in accordance to ethical obligations to the patients and to the applicable consent agreement?	Not Applicable	
Are **computational models** that are central and integral to a study available without restrictions in a machine-readable form? Were the relevant accession numbers or links provided?	Not Applicable	
If publicly available data were reused, provide the respective **data citations in the reference list**.	Not Applicable	
